# Intracerebral Hemorrhage: Advances, Knowledge Gaps, and Future Directions

**DOI:** 10.1002/mco2.70436

**Published:** 2025-10-26

**Authors:** Tao Liu, Weiwei Jiang, Minzhi Zhang, Shangzhi Xiong, Linan Chen, Xiaoying Chen, Rongcai Jiang

**Affiliations:** ^1^ Department of Neurosurgery Xuanwu Hospital Capital Medical University Beijing China; ^2^ Department of Rehabilitation Medicine Zhujiang Hospital of Southern Medical University Guangzhou Guangdong China; ^3^ Department of Neurology Tianjin Medical University General Hospital Tianjin China; ^4^ The George Institute For Global Health Sydney New South Wales Australia

**Keywords:** intracerebral hemorrhage, management, neural repair and recovery, neuroimaging, pathophysiology

## Abstract

Acute spontaneous intracerebral hemorrhage (ICH) remains a severe and challenging cerebrovascular condition, associated with high rates of morbidity and mortality. The pathophysiology of injury following ICH involves mass effect, increased intracranial pressure, hematoma expansion, and toxicity from blood‐breakdown products. Over the past decade, substantial progress has been made in risk stratification, therapeutic strategies, and outcome prognostication. Although case‐fatality rates have declined with advances in neuroimaging, acute care, and surgical techniques, functional outcomes remain poor, with little improvement. Several trials have investigated the optimal medical and surgical treatments for ICH, but none have shown significant improvements in outcomes or survival. This review aims to provide a comprehensive overview of ICH, including its epidemiology, associated costs, pathophysiological mechanisms, and management approaches. Herein, we explored recent advancements in neuroimaging techniques and their roles in diagnosing ICH and predicting patient outcomes. Additionally, we assessed the impact of prehospital and in‐hospital management practices, such as pharmacological and surgical interventions, and discussed the implications of delays before final treatment. By summarizing current research and evidence‐based practices, this review aims to highlight established and emerging strategies for improving outcomes for patients with ICH and identify areas for future research and development in the field.

## Graphical abstract

This review provides a comprehensive synthesis of current research on the epidemiology, pathophysiological mechanisms, imaging characteristics, treatment delays, clinical management strategies, neural repair, and prognostic prediction of intracerebral hemorrhage (ICH). The findings contribute to the development of evidence‐based strategies and innovative approaches aimed at improving patient outcomes, while also offering clear directions for future research in this field.

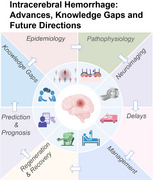

## 1 Introduction

Spontaneous intracerebral hemorrhage (ICH) is a common and severe form of stroke associated with high mortality rates and frequent motor and other neurological deficits. It is defined as nontraumatic bleeding within the brain parenchyma, most often caused by rupture of small penetrating arterioles from hypertensive arteriopathy or cerebral amyloid angiopathy (CAA). The hemorrhage typically forms a parenchymal hematoma that may expand rapidly and can extend into the ventricular system; limited subarachnoid extension can also occur. As a global public health issue, ICH carries a significantly higher disability burden—approximately 50% greater than ischemic stroke—and displays notable regional variability, particularly affecting low‐ and middle‐income countries (LMICs) [1, 2]. This variability highlights the heterogeneous nature of ICH, which is influenced by its diverse causes, clinical presentations, and prognostic outcomes [3].

Epidemiological studies reveal a high incidence of ICH, with prevalence varying according to geographic, demographic, and socioeconomic factors [1]. The healthcare burden is substantial, encompassing direct medical costs and indirect costs related to long‐term disability and loss of productivity [1]. The sudden onset of ICH often leads to rapid neurological deterioration, with approximately one‐third of patients dying within the first month [3]. Survivors frequently experience varying degrees of disability and experience an increased risk of recurrent hemorrhage, severe vascular events, and neurological complications, such as seizures and dementia. Therefore, timely medical intervention is critical for improving patient outcomes.

**FIGURE 1:**
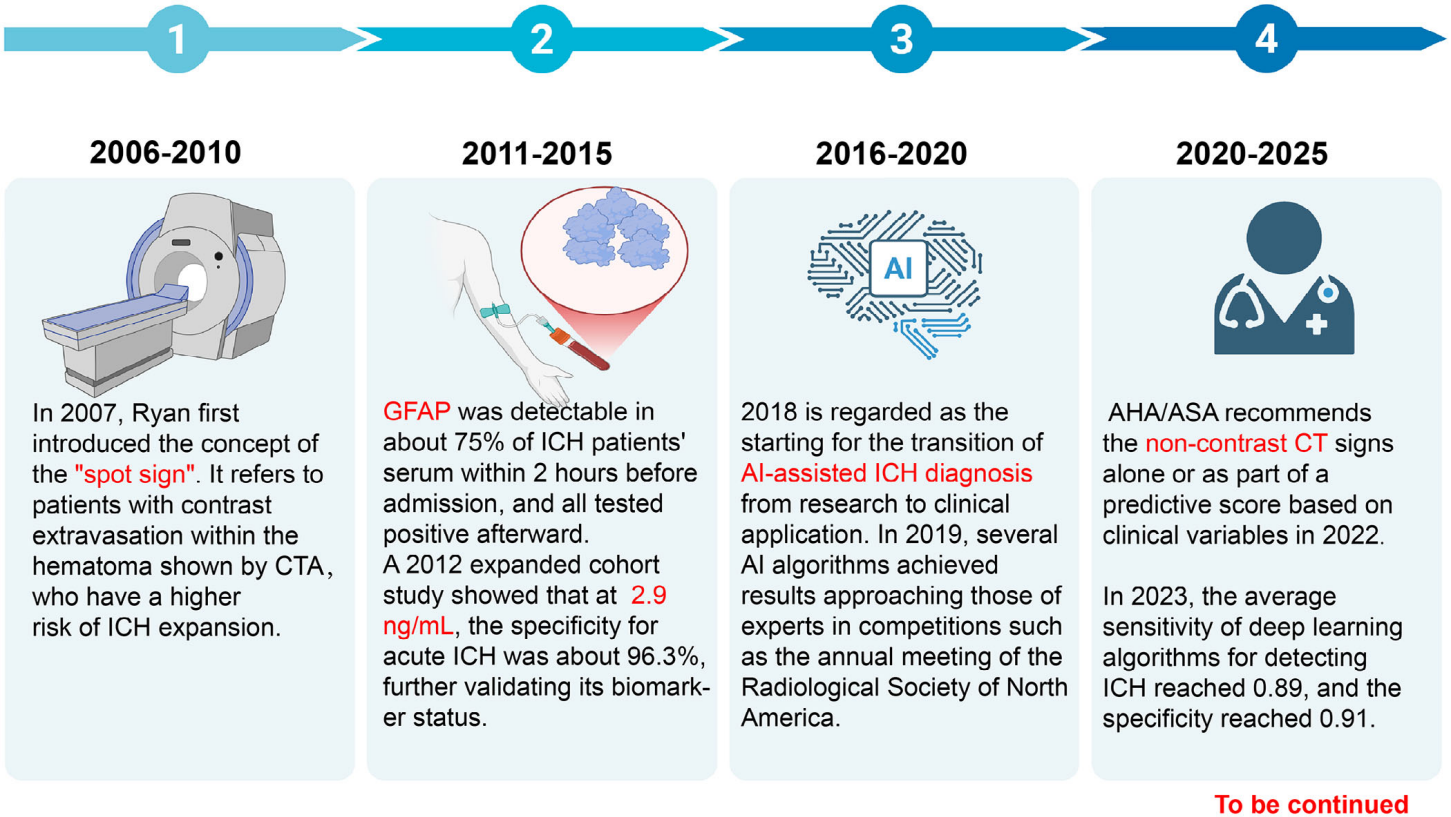
Advances in diagnostic techniques for intracerebral hemorrhage. In 2007, the “spot sign” was first proposed for predicting the risk of HE. In 2010, the AHA/ASA guidelines were updated to recommend CTA and contrast‐enhanced CT for identifying the risk of HE (Class IIb, Level B evidence). Serum GFAP can be detected in approximately 75% of patients with ICH within 2 h before admission, and subsequently nearly in all patients due to acute ICH‐induced sudden disruption of the BBB and rapid damage to astrocytes. AI‐based imaging interpretation technology plays a crucial role in stroke diagnosis, enabling automated ICH detection, differentiation of hemorrhage subtypes. Additionally, it has the ability to detect skull fractures, midline shift, and the mass effect of hematomas. AHA, American Heart Association; AI, artificial intelligence; ASA, American Stroke Association; BBB, blood–brain barrier; CT, computerized tomography; CTA, CT angiography; GFAP, glial fibrillary acidic protein; HE, hematoma expansion; ICH, intracerebral hemorrhage.

Advances in neuroimaging and molecular diagnostics, along with a growing understanding of ICH pathophysiology and neural repair processes have led to improved diagnostic and therapeutic capabilities for ICH (Figure 1). Among these advancements, blood‐based biomarkers have been investigated as tools for early differentiation between ischemic and hemorrhagic stroke. As early as 2006, glial fibrillary acidic protein (GFAP) was identified as an important blood‐based biomarker for hemorrhagic stroke [4]. In an expanded cohort, a GFAP threshold of approximately 2.9 ng/mL yielded ≈96% specificity for acute ICH, although performance varies by assay and sampling time [5]. Combining plasma with GFAP has been reported to improve the accuracy of classifying stroke types [6]. Neuroimaging remains the standard tool for determining stroke subtype, and identifying patients at a risk of hematoma expansion (HE) has been a central focus in neuroimaging research. In 2007, the computed tomographic angiography (CTA)‐based “spot sign”—contrast extravasation within the hematoma—was first described as a predictor of HE [7]. Since around 2018, deep learning tools for automated ICH detection and subtype classification have gained traction for clinical evaluation; subsequent algorithmic refinements using large, multi‐institutional datasets have further improved diagnostic sensitivity and specificity [8, 9, 10].

Notable advances have been made in ICH prevention strategies, particularly in reducing HE, with early blood pressure (BP) management showing promise [11, 12]. However, ICH remains to a challenging condition due to its unpredictable and diverse causes, as well as the subsequent need for personalized care strategies. Moreover, current pharmacological and surgical treatments, despite some short‐term effects, have yet to show significantly improvements in patients' long‐term functional outcomes [13]. New developments in care bundle approaches offer hope for better results, though further research is needed to refine these approaches for treatment [14]. Stem cell therapy has the potential to promote nerve regeneration. Its clinical exploration for stroke treatment began as early as 2009 [15], progressed to clinical studies in 2013, and advanced to randomized controlled studies in 2017 [16, 17]. However, the number and scale of stem cell clinical trials for ICH are relatively limited compared with those for ischemic stroke. In summary, despite significant progress in understanding the risks, diagnosis, and treatment methods over the past two decades, ICH remains a challenging clinical condition. The medical community urgently needs to conduct in‐depth analyses of the pathological mechanisms, epidemiological characteristics, and clinical manifestations of ICH. Further research is needed to continue improving early diagnosis and treatment strategies, with a particular emphasis on innovative therapies and support for LMICs, where resources are more limited.

In this review, we comprehensively evaluated the current international evidence on ICH, including its epidemiological characteristics, pathological mechanisms, imaging findings, and clinical management methods. Our aim was to generate theoretical support for prevention and therapeutic practices of ICH, as well as to highlight implications for further research. Recent advancements offer promising insights into reducing brain injury, preventing complications, and facilitating the recovery of this complex and challenging condition.

## 2 Epidemiology and Economic Burden

Epidemiological factors influence the incidence and mortality rates of ICH. In 2020, nearly 12 million new‐onset stroke cases were reported worldwide, with ICH accounting for approximately 30% of these cases. The incidence of ICH is particularly high in Southeast Asia and parts of sub‐Saharan Africa [18]. In the United States, the age‐ and sex‐adjusted incidence of primary ICH increased by approximately 11% between 2004 and 2018 [19]. In contrast, China has seen a gradual decline in the incidence of ICH over the past decade, dropping from 61 per 100, 000 in 2010 to 45 per 100, 000 in 2019 [20]. However, these rates remain significantly higher than those in high‐income countries. From 1990 to 2021, the top three countries with the largest increases in the age‐standardized incidence of ICH were Lesotho, Philippines, and Uzbekistan, all of which are LMICs, whereas the countries with the largest decreases were South Korea, Portugal, and Estonia, all of which are high‐income countries [1]. Notably, the incidence of new ICH cases in LMICs is nearly double that of high‐income countries, underscoring the strong correlation between the incidence of ICH and a country's economic status and level of development.

**FIGURE 2:**
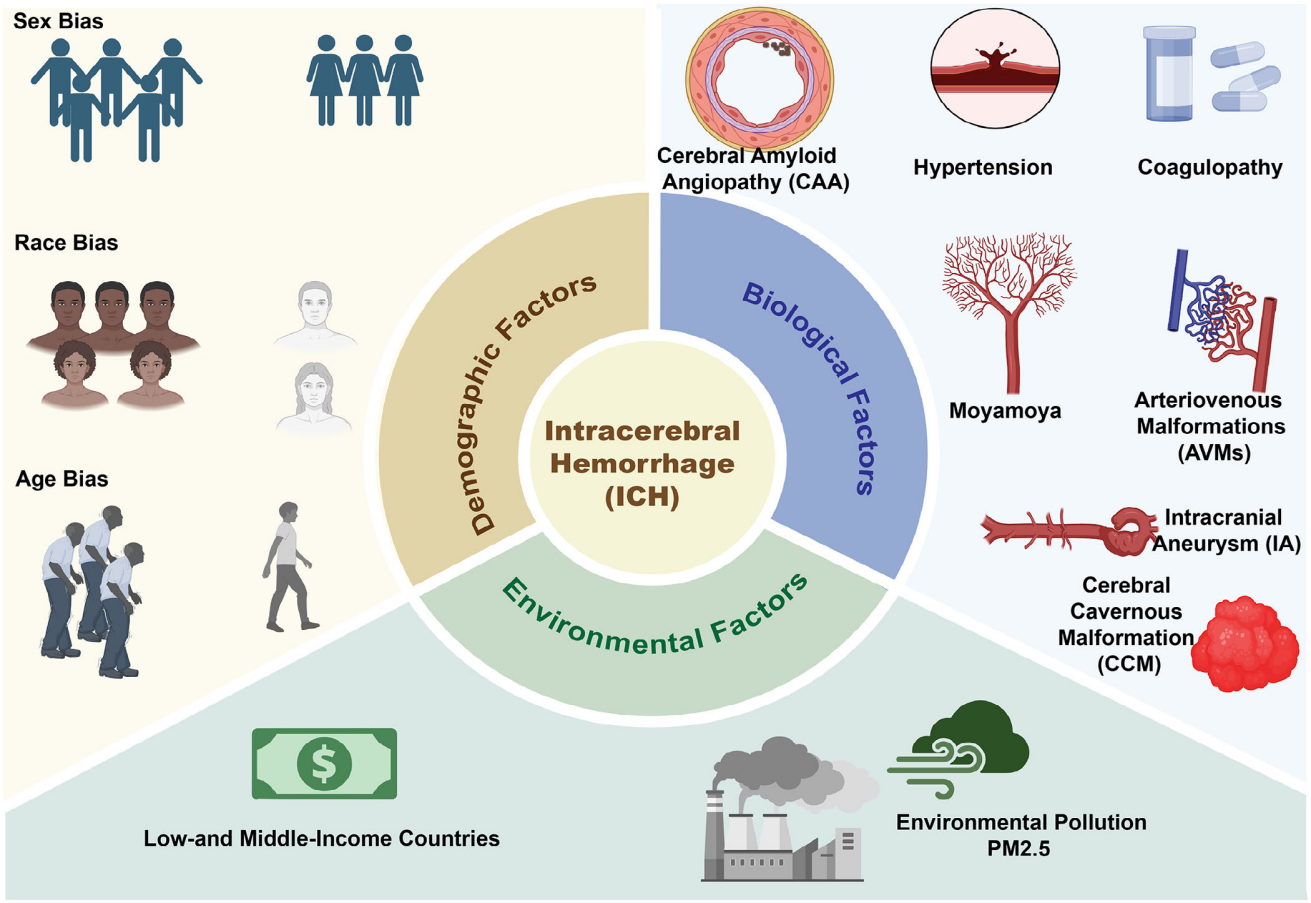
Epidemiological factors contributing to intracerebral hemorrhage. The risk of ICH is influenced by biological factors (e.g., chronic hypertension, arteriovenous malformations, and aneurysms), demographic factors (such as sex, race, and age), and environmental factors.

Various risk factors have been found to be significantly associated with ICH, including biological factors, demographic factors, and environmental factors (Figure 2). Major biological risk factors for ICH include hypertension‐induced microvascular damage, CAA, and coagulopathy [21]. Of note, recent studies indicate that proactive BP management and antiplatelet therapy can reduce the risk of ICH recurrence and improve functional outcomes at 6 months; however, the potential increase in major adverse cardiovascular events associated with antithrombotic therapy in patients with ICH remains unclear [11, 12, 14, 22, 23]. Various demographic factors such as sex, age, and race also contribute to varying trends in the incidence of ICH. Between 1990 and 2013, the global incidence of ICH in individuals aged 20–64 years nearly doubled, with a significant rise also observed in those under 20 years [24, 25]. This trend toward earlier onset of ICH could result in long‐term disability, high healthcare costs, and economic productivity losses, highlighting its substantial public health impact. Additionally, Black and Hispanic individuals have a higher risk of ICH, earlier onset, and a greater likelihood of ischemic stroke following ICH compared with Caucasians [26, 27]. These disparities may stem from differences in hypertension management adequacy, lifestyle factors, healthcare access, and biological differences. Moreover, the global incidence of ICH has increased over the past 30 years, which was attributed by some researchers to environmental factors, primarily the high exposure to PM2.5, particularly among older men and in low‐income countries [28]. Notably, while ICH rates linked to household air pollution have decreased in many regions, notable increases have been reported in Zimbabwe and the Philippines [29].

ICH, with its high fatality rate, imposes a substantial health burden globally. Statistics show that approximately one‐third of patients with ICH die within the first month of onset, and only half survive beyond 1 year [3]. Those who do survive often experience serious neurological complications, including recurrent ICH, systemic vascular diseases, epilepsy, and cognitive impairments, complicating stroke management [3, 30, 31]. In 2019, ICH accounted for approximately 3 million deaths worldwide and 69 million disability‐adjusted life years, surpassing the burden of ischemic stroke [1]. Using the value of lost welfare (VLW) method, which combines gross domestic product (GDP) and disability‐adjusted life year data, the global VLW attributed to ICH in 2019 was estimated at $883 billion or 0.71% of global GDP. East Asia, Southeast Asia, and Oceania exhibited the highest VLW, whereas high‐income regions had the lowest VLW‐to‐GDP ratio, underscoring that LMICs continue to bear the brunt of the global ICH burden [32].

The economic burden of ICH is significant. A study in Canada found that the median hospitalization cost for patients with ICH was roughly ten times higher than that for other patients, with an average annual cost of $48, 549 for survivors—far exceeding the cost for those who died during hospitalization [33]. These expenses are driven by the need for long‐term oral anticoagulant (OAC) therapy, ongoing care, and rehabilitation services. According to a 2019 survey, the number of hospitalized patients with ICH in China reached 611, 709, and the per capita hospitalization cost was 20, 106 yuan, which doubled compared with that of the past decade [20]. This increase reflects not only the aging population and widespread risk factors but also growing public awareness of stroke treatment and prevention of recurrence.

## 3 Pathophysiology and Animal Models

**FIGURE 3:**
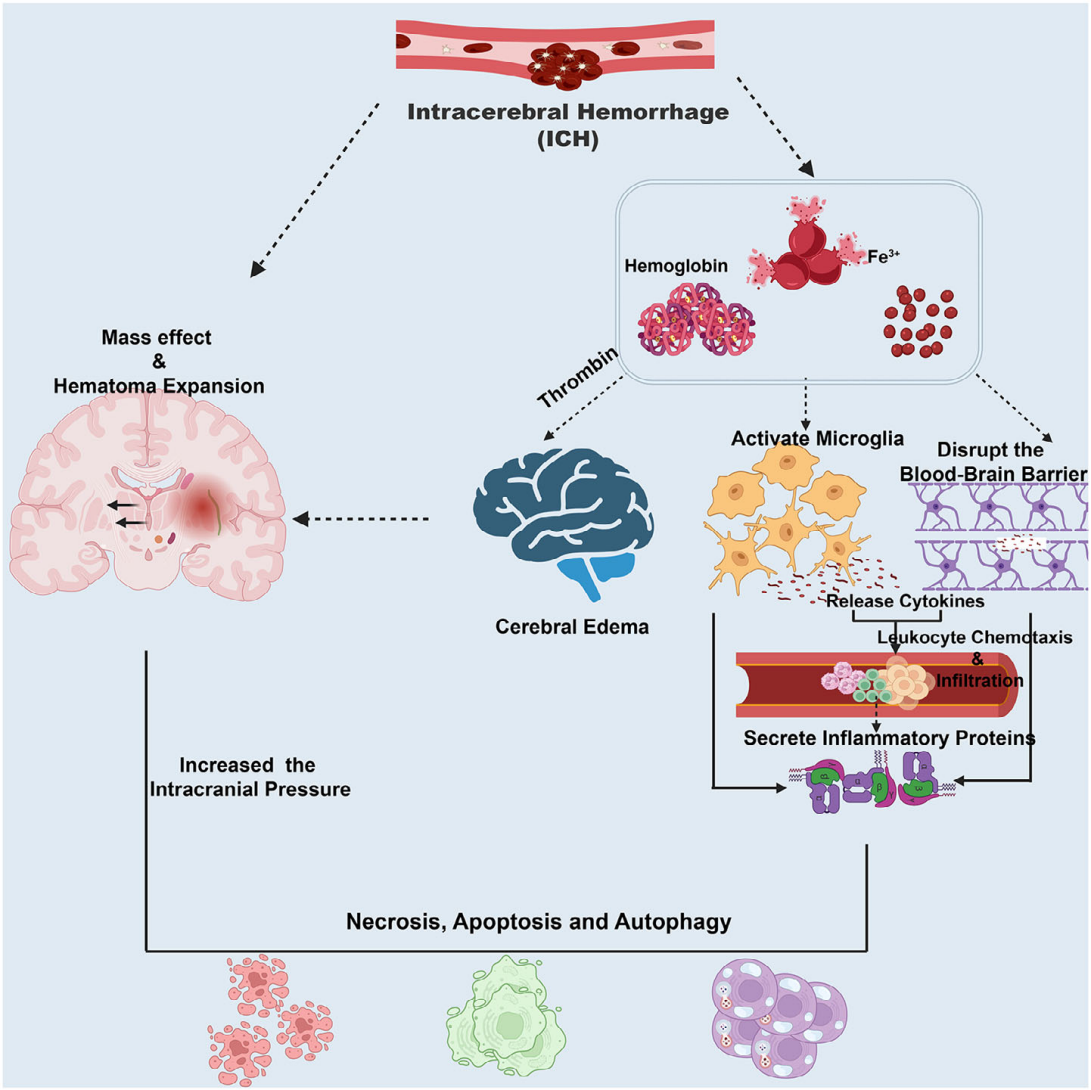
Mechanisms of brain injury in intracerebral hemorrhage. Primary brain injury in ICH results from direct neuronal damage caused by the hematoma, which alters ICP and deforms local structures depending on the hemorrhage's size and location. HE from ongoing bleeding is a major risk factor that exacerbates brain injury. The initial mass effect of the hematoma may shear surrounding small arteries, leading to secondary bleeding and re‐expansion of the hematoma, thereby contributing to neuronal injury and necrosis. Secondary brain injury stems from the brain's pathological response to the hematoma's toxic components and metabolic effects. Oxidative stress from toxic blood components leads to brain edema, disruption of the BBB, immune activation, neuroinflammation, and neuronal death. BBB, blood–brain barrier; HE, hematoma expansion; ICH, intracerebral hemorrhage; ICP, intracranial pressure.

Although the sites predisposed to ICH vary depending on the etiology, the pathological mechanisms of nerve damage caused by the hemorrhage share common features. In this section, we have explored the general pathological mechanisms of ICH (Figure 3).

The brain injury following ICH can be categorized into two types—primary and secondary. Primary brain injury in ICH refers to direct neuronal damage caused by the hematoma, which alters intracranial pressure (ICP) and deforms local structures depending on the hemorrhage's size and location. HE due to ongoing bleeding is a major risk factor that exacerbates brain injury [34]. The space‐occupying effects of the hematoma contribute to neuronal injury and necrosis. Efforts to mitigate primary brain injury focus on preventing HE by controlling BP, correcting coagulopathy, or surgically removing or dissolving the blood clot through open or minimally invasive procedures [35].

Secondary brain injury stems from the brain's pathological response to the hematoma's toxic components and metabolic effects, leading to severe neurological deficits. Hemolysis of red blood cells begins approximately 24 h after ICH, continues for several days, and contributes to brain injury through the release of cytotoxic hemoglobin [36]. Hemoglobin and its breakdown products (heme and iron) generate oxidative stress by producing free radicals that damage proteins and nucleic acids, disrupt sodium–potassium pumps, and impair the exchange of substances inside and outside cells, causing osmotic imbalances [37, 38, 39]. Experimental ICH models have demonstrated that oxidative stress from toxic blood components leads to brain edema, disruption of the blood–brain barrier (BBB), immune activation, neuroinflammation, and neuronal death [37, 39].

Brain edema is the most immediate and severe secondary injury following ICH. Clinical studies show that within 24 h of acute ICH, the volume of perihematomal edema (PHE) can increase by approximately 75% [40]. The formation of edema after ICH is complex. In animal models, vasogenic edema formation is associated with thrombin, which is rapidly activated after brain hemorrhage and plays a role in both hemostasis and disruption of endothelial cells and the BBB [41, 42]. High concentrations of thrombin have been shown to induce neuronal damage in vitro, whereas low concentrations offer neuroprotection against ischemic or oxidative stress [43].

Microglia and astrocytes are the first immune cells to respond to blood extravasation. In ICH mice models, M1 proinflammatory microglia rapidly increase within 6 h of injury and decline over the next 14 days, whereas M2 anti‐inflammatory microglia are initially low but increase progressively during this period [44, 45]. Astrocytes regulate microglial polarization and transportation of water and other molecules from blood vessels to brain tissue, potentially causing brain edema and BBB damage after hemorrhage [46]. Activated microglia release cytokines and chemokines that recruit peripheral immune cells through the damaged BBB to the site of hemorrhage [44].

Neutrophils, one of the first peripheral white blood cells to infiltrate the brain after ICH, contribute to injury by producing reactive oxygen species and proinflammatory proteins, further disrupting the BBB. Animal studies show that depleting neutrophils before ICH offers neuroprotection, while depleting them after ICH worsens neuronal damage, indicating neutrophil's complex role in ICH pathology [47, 48]. Infiltrating macrophages, which have strong erythrophagocytic capabilities, express higher levels of major histocompatibility complex II than microglia [49]. Depletion of Ly6Chi monocytes has been shown to resolve brain injury and improve neurological function in ICH mouse models, suggesting their involvement in post‐ICH neuroinflammation [50].

While CD8^+^ cytotoxic T cells and CD4^+^ helper T cells infiltrate brain tissue within days following ICH, their specific roles remain unclear [51, 52]. Using single‐cell transcriptomics, Li et al. [53] revealed that natural killer (NK) cells infiltrate the brain early following ICH, exacerbating BBB disruption and brain edema by killing endothelial cells and recruiting neutrophils. As the innate immune response activates and peripheral immune cells infiltrate, various inflammatory mediators—including chemokines, tumor necrosis factors (TNFs), adhesion molecules, and matrix metalloproteinases (MMPs)—contribute to local cell death associated with neuroinflammation [54, 55]. Neuronal death after ICH occurs via necrosis and apoptosis, though the dominant process remains debated. In a study of tissue samples from 12 patients with ICH who underwent surgical hematoma evacuation, Qureshi et al. [56] found that 25% of cells in the perilesional area exhibited necrosis. Necrosis leads to cell swelling and the release of intracellular components, provoking significant inflammatory responses. Apoptosis, characterized by membrane shrinkage and nuclear condensation, peaks 24 h after ICH, with fewer neurons remaining afterward [57]. Additionally, HE and tissue compression post‐ICH induce hypoxia, which can trigger autophagy [58]. Over time, autophagy plays a dual role, contributing to neuroinflammation and tissue repair [59, 60, 61].

## 4 Landmark Clinical Trials and Current Evidence

Recent research has explored a spectrum of therapeutic strategies for ICH, including early hemostatic therapy to limit hematoma growth, intensive BP lowering treatment, surgical and minimally invasive clot evacuation techniques, neuroprotective interventions, and prevention and management of complications. As summarized in Table 1, randomized controlled trials (RCTs) across these domains provide evidence to guide clinical decision‐making, though effects on functional outcomes have been inconsistent. The risk of bias was assessed using the Cochrane RoB2 tool.

**TABLE 1:** Summary of RCTs on ICH.

Reference	Settings	RoB2	Inclusion criteria	No.	Intervention	Primary outcomes	Study status	Key results
INTERACT [12]	Multicenters (44 centers, 3 countries)	Low	Acute spontaneous ICH within 6 h, aged ≥18 years, elevated SBP 150–220 mmHg	404	Intensive BP reduction target <140 mmHg vs. guideline target <180 mmHg	Hematoma growth at 24 h	Complete	Intensive BP‐lowering treatment reduced hematoma growth in ICH but did not improve functional outcomes.
INTERACT2 [11]	Multicenter (144 centers, 21 countries)	Low	Spontaneous ICH within 6 h, aged ≥18 years, SBP >150 mmHg, GCS >5	2839	Intensive BP reduction target <140 mmHg vs. guideline target <180 mmHg	mRS at 90d	Complete	Intensive BP lowering improved functional outcomes.
INTERACT3 [14]	Multicenter (121 centers, 10 countries)	Some concerns	Spontaneous ICH within 6 h, aged ≥18 years	7036	Care bundle group vs. usual bundle group	mRS at 6 months	Complete	A care bundle for intensive BP lowering improved functional outcome.
INTERACT4 [62]	China (51 centers)	Low	Suspected stroke within 2 h, aged ≥18 years, SBP ≥150 mmHg	2404	Intensive prehospital BP lowering target 130–140 mmHg vs. standard care	mRS at 90d	Complete	Early BP reduction improved outcomes in ICH but worsened outcomes in IS.
ATACH‐2 [63]	Multicenter (110 centers, 5 countries)	Low	Spontaneous ICH within 4.5 h, aged ≥18 years, HV <60 cm^3^, GCS ≥5	1000	Intensive BP reduction target 110–139 mmHg vs. guideline target 140–179 mmHg	mRS at 3 months	Complete	Intensified BP lowering did not improve functional outcomes but increased the incidence of adverse events.
RIGHT‐2 [64]	UK (54 centers)	Low	Presumed stroke within 4 h of onset, aged ≥18 years, SBP ≥120 mmHg	1149	Transdermal GTN 5 mg/d for 4d vs. sham group (similar sham dressing)	mRS at 90d	Complete	Prehospital transdermal GTN did not improve functional outcomes in presumed stroke.
MR ASAP [65]	Netherlands (6 centers)	Some concerns	Acute stroke within 3 h, aged ≥18 years, SBP ≥140 mmHg	325	Transdermal GTN 5 mg/day for 24 h plus standard care vs. standard care alone	mRS at 90d	Complete	Early transdermal GTN is ineffective and should be avoided in acute ICH.
STOP‐MSU [66]	Multicenter (25 centers, 5 countries)	Low	Acute spontaneous ICH within 2 h, aged ≥18 years, HV ≤70 mL, GCS ≥8	201	TXA 1 g over 10 min followed by 1 g over 8 h iv vs. placebo	Hematoma growth within 24 h	Complete	Intravenous TXA administered within 2 h of ICH onset did not reduce hematoma growth or affect functional outcome.
FAST‐MAG [67]	USA (60 centers)	Low	Suspected stroke within 2 h, aged 40–95 years	1700	Mg sulfate 4 g over 15 min followed by 16 g over 24 h iv vs. placebo	mRS at 3 months	Complete	Prehospital initiation of Mg sulfate therapy did not improve functional outcomes.
CHASE [68]	China (26 centers)	Low	Acute stroke within 72 h, aged ≥18 years, GCS ≥12, NIHSS ≥11, SBP 150–210 mmHg	483	Individualized group (10–15% SBP reduction) vs. standard group (target SBP <200 mmHg for IS and <180 mmHg for ICH	Proportion of poor functional outcome at 90d	Complete	Individualized BP lowering treatment did not reduce death or disability in acute severe stroke patients.
Dong et al. [69]	China (14 centers)	Some concerns	Acute ICH within 24 h, aged ≥18 years, SBP ≥150 mmHg	338	Standard guideline management with remifentanil and dexmedetomidine vs. standard guideline management	SBP control rate (less than 140 mmHg) at 1 h posttreatment initiation	Complete	The intervention improved SBP control at 1 h but had no effect on functional outcomes or hematoma growth.
Mayer et al. [70]	Multicenter (73 centers, 20 countries)	Low	Spontaneous ICH within 3 h, age ≥18 years	399	rFVIIa 40/80/160 µg/kg iv vs. placebo	Percent change in ICH volume at 24 h	Complete	rFVIIa reduced hematoma growth, lowered mortality, and improved functional outcomes.
FAST [71]	Multicenter (122 centers, 22 countries)	Low	Spontaneous ICH within 3 h, age ≥18 years	841	rFVIIa 20/80 µg/kg iv vs. placebo	mRS at 90d	Complete	rFVIIa reduced hematoma growth but did not improve functional outcomes.
PATCH [72]	Multicenter (60 centers, 3 countries)	Low	Nontraumatic, supratentorial ICH within 6 h, aged ≥18 years, GCS ≥8	190	Platelet transfusion vs. standard care	mRS at 3 months	Complete	Platelet transfusion is associated with worse functional outcomes.
MACH [73]	Canada (1 center)	Some concerns	Primary ICH within 24 h, aged ≥18 years, GCS ≤5	16	Minocycline 400 mg iv followed by 400 mg oral daily for 4d vs. placebo	mRS at 90d	Complete	A 400 mg dose of minocycline was safe and achieved neuroprotective serum concentrations.
TICH‐2 [74]	Multicenter (124 centers, 12 countries)	Low	Acute ICH within 8 h, aged ≥18 years, GCS ≥5	2325	TXA 1 g over 10 min followed by 1 g over 8 h iv vs. placebo	mRS at 90d	Complete	TXA did not improve functional outcomes.
Selim et al. [75]	Multicenter (40 centers, 2 countries)	Low	Spontaneous, supratentorial ICH within 24 h, age 18–80 years	291	DFO (32 mg/kg/day) for 3d vs. placebo	mRS (0–2) at 90d	Complete	DFO did not improve functional outcomes.
RESTART [23]	UK (122 centers)	Some concerns	Spontaneous ICH, aged ≥18 years, taking antithrombotic therapy	537	Start vs. avoid antiplatelet therapy	Recurrent symptomatic ICH for up to 5 years	Complete	Antiplatelet therapy did not increase the risk of recurrent hemorrhage.
SPOTLIGHT‐STOP‐IT [76]	Multicenter (26 centers, 2 countries)	Low	Acute, spontaneous ICH within 3 h, aged ≥18 years, a spot sign on CT	69	rFVIIa 80 µg/kg iv vs. placebo	Hematoma growth at 24 h	Complete	rFVIIa did not improve radiographic or clinical outcomes.
STOP‐AUST [77]	Multicenter (13 centers, 3 countries)	Low	Nontraumatic ICH within 4.5 h, aged ≥18 years, with a spot sign on CT	100	TXA 1 g over 10 min followed by 1 g over 8 h iv vs. placebo	Hematoma growth at 24 h	Complete	TXA did not reduce hematoma growth or mortality.
EFFECTS [78]	Sweden (35 centers)	Low	Stroke, aged ≥18 years, at least one persisting focal neurological deficit	1500	Fluoxetine 20 mg oral once daily for 6 months vs. placebo	mRS at 6 months	Complete	Fluoxetine did not improve functional outcomes and increased the risk of bone fractures and hyponatremia.
Takeuchi et al. [79]	Japan (1 center)	Some concerns	SAH within 72 h, aged 20–80 years, ruptured aneurysm	37	Mg vs. H_2_+Mg vs. placebo	Occurrence of DCI, CVS	Complete	Intracisternal Mg sulfate infusion after surgery reduces CVS and DCI.
GATE‐ICH [80]	China (26 centers)	Some concerns	Acute ganglia ICH within 72 h, aged ≥18 years, HV 5–30 mL, GCS ≥6	200	Glibenclamide orally (1.25 mg, 3/day) plus standard care vs. standard care	mRS at 90d	Complete	Glibenclamide did not reduce the proportion of poor outcome.
TICH‐NOAC [81]	Switzerland (6 centers)	Low	Acute nontraumatic ICH within 12 h, aged ≥18 years	63	TXA 1 g over 10 min followed by 1 g over 8 h iv vs. placebo	HE	Complete	Early administration of TXA (<6 h) could reduce HE.
DASH [82]	UK (10 centers)	Low	Spontaneous ICH within 12 h, aged ≥18 years, oral antiplatelet drug	54	Desmopressin 20 µg iv vs. placebo	mRS at 90d	Complete	Desmopressin was feasible in spontaneous ICH patients on antiplatelets.
Connolly et al. [83]	Multicenter (131 centers, 23 countries)	Some concerns	Acute ICH within 6 h, use of Xa inhibitors within 15 h, GCS ≥7	452	Andexanet vs. usual care	Effective hemostasis	Complete	Andexanet reduced hematoma expansion but increased IS risk, without reducing mortality.
STICH [84]	Multicenter (83 centers, 27 countries)	Some concerns	Spontaneous supratentorial ICH within 72 h, hematoma ≥2 cm, GCS ≥5	1033	Early surgery combined hematoma evacuation (within 24 h) vs. medical treatment	Eight‐point GOS at 6 months	Complete	Early surgery did not improve the prognosis.
STICH II [85]	Multicenter (78 centers, 27 countries)	Low	Lobar ICH within 48 h (10–100 mL, ≤1 cm from cortex), GCS motor score 5–6, eye score ≥2	601	Early surgery combined hematoma evacuation vs. medical treatment	Eight‐point GOS at 6 months	Complete	Early surgery did not improve the prognosis.
MISTIE [86]	Multicenter (26 centers, 4 countries)	Some concerns	Spontaneous, supratentorial ICH within 12 h (≥20 mL), age 18–80 years, GCS ≤14 or NIHSS ≥6, HE <5 mL after 6 h	96	MISTIE (image‐guided minimally invasive surgery + 0.3 mg or 1 mg alteplase every 8 h for up to 9 doses) vs. standard medical care	mRS at 180d	Complete	MIS plus rt‐PA is safe but increases the risk of asymptomatic hemorrhage.
MISTIE III [87]	Multicenter (78 centers, 9 countries)	Low	Spontaneous, supratentorial ICH (≥30 mL), aged ≥18 years, GCS ≤14 or NIHSS ≥6, HE <5 mL after 6 h	506	MISTIE (image‐guided minimally invasive surgery +1.0 mg alteplase every 8 h for up to nine doses) vs. standard medical care	mRS at 365d	Complete	For moderate to large ICH, MISTIE did not improve functional outcomes.
MISICH [88]	China (14 centers)	Some concerns	Supratentorial ICH within 24 h (≥25 mL), age 18–80 years, GCS ≥5	733	Endoscopic surgery vs. stereotactic aspiration vs. craniotomy	mRS at 6 months	Complete	Endoscopic surgery and stereotactic aspiration improved the long‐term outcome of hypertensive ICH, especially supratentorial, deep hemorrhage.
SWICH [89]	Multicenter (42 centers, 9 countries)	Low	Supratentorial, deep ICH within 66 h (30–100 mL), aged 18–75 years, GCS 8–13	197	DC plus best medical treatment vs. best medical treatment alone	mRS at 6 months	Complete	DC plus best medical treatment did not improve functional outcomes.
Pradilla et al. [90]	USA (37 centers)	Low	Supratentorial, spontaneous, acute ICH within 24 h (30–80 mL); aged 18–80 years; GCS 5–14	300	Minimally invasive hematoma removal plus guideline‐based medical management vs. medical management alone	mRS at 180d	Complete	Minimally invasive hematoma evacuation improved functional outcomes.
CARICH [91]	China (1 center)	Some concerns	Supratentorial ICH within 72 h (30–80 mL), aged 18–80 years, GCS ≥5	204	DC plus clot removal vs. clot removal alone	mRS at 3 months	Complete	Clot removal without DC improved functional outcomes and reduced mortality.
Wang et al. [92]	China (1 center)	Some concerns	First‐ever unilateral stroke in the middle cerebral artery territory within 2 weeks to 3 months, aged 30–85 years, initial FMA score <50/100	45	10 Hz rTMS (HF group) vs. 1 Hz rTMS (LF group) vs. sham rTMS (sham group) daily for 2 weeks	FMA score at 2 weeks	Complete	HF‐rTMS over the contralesional cortex was superior to low‐frequency rTMS and sham stimulation for motor recovery.
PEACH [93]	France (3 centers)	Low	Nontraumatic, supratentorial ICH within 24 h, aged ≥18 years, NIHSS ≤25	50	Levetiracetam (500 mg every 12 h) iv vs. placebo	At least one clinical or electrographic seizure within 72 h	Complete	Levetiracetam might be effective in preventing acute seizures.
Vink et al. [94]	Netherlands (1 center)	Low	First‐ever stroke within 3 weeks, aged ≥18 years, paresis of 1 arm (motricity index 9–99)	60	Contralesional cTBS vs. sham cTBS, 10 daily sessions over 2 weeks	Change in action research arm test score at 3 months poststroke	Complete	Contralesional cTBS promote recovery of the upper limb and reduce disability.

Abbreviations: BP, blood pressure; cTBS, continuous theta‐burst stimulation; CVS, cerebral vasospasm; DC, decompressive craniectomy; DCI, delayed cerebral ischemia; DFO, deferoxamine; FMA, Fugl‐Meyer motor assessment; GCS, Glasgow coma scale; GOS, Glasgow outcome scale; GTN, glyceryl trinitrate; HE, hematoma expansion; HV, hematoma volume; ICH, intracerebral hemorrhage; IS, ischemic stroke; iv, intravenous; IVH, intraventricular hemorrhage; Mg, magnesium; mRS, modified Rankin scale; NIHSS, National Institutes of Health Stroke Scale; rFVIIa, recombinant activated factor VII; rt‐PA, recombinant tissue plasminogen activator; SAH, subarachnoid hemorrhage; SBP, systolic blood pressure; SET, stroke‐related early tracheostomy; tTMS, repetitive transcranial magnetic stimulation; TXA, tranexamic acid; UK, United Kingdom; USA, United States of America.

## 5 Neuroimaging and Diagnostic Advances

**FIGURE 4:**
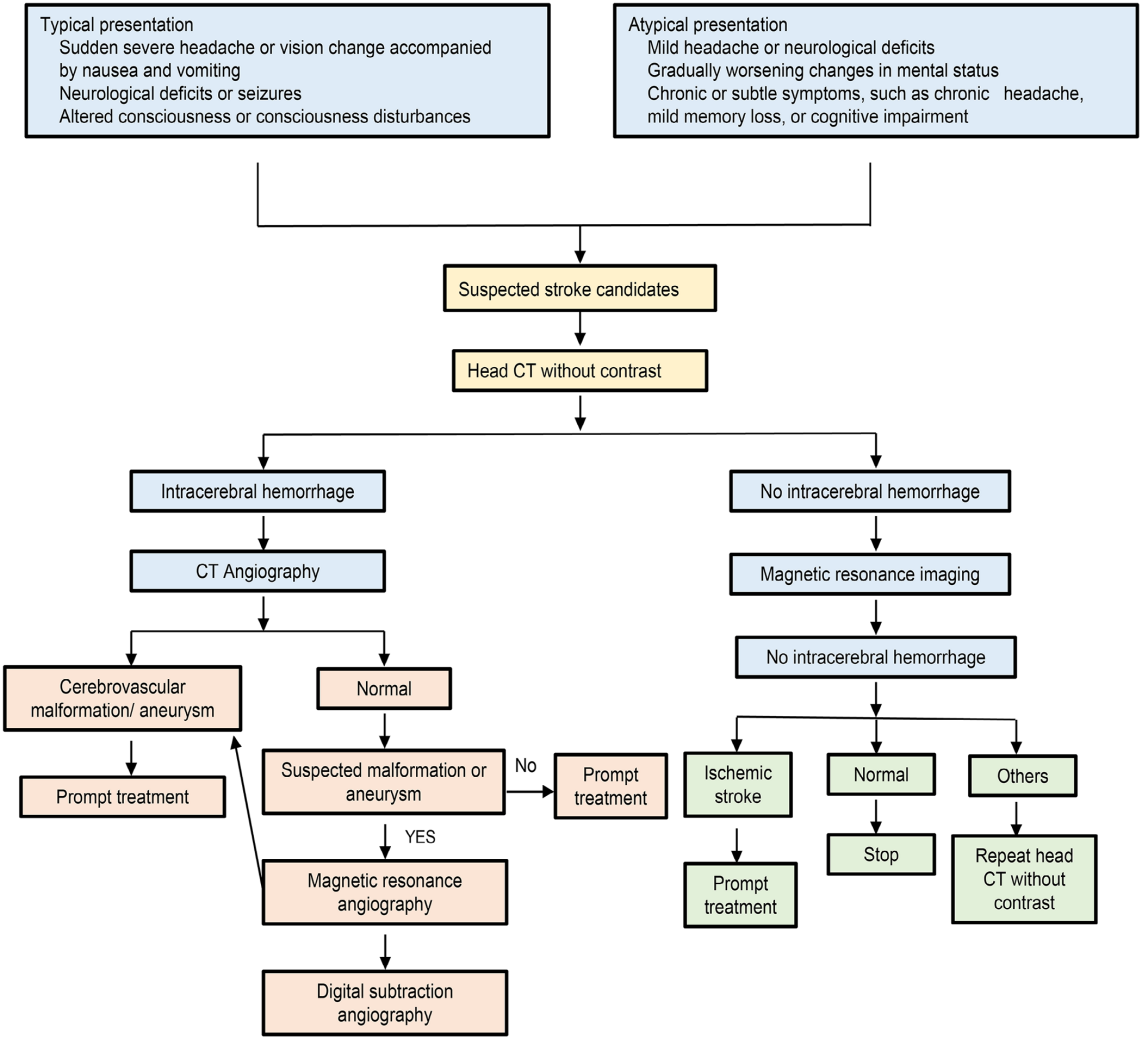
Diagnostic algorithm for intracerebral hemorrhage. For patients presenting with symptoms, a noncontrast head CT scan is initially performed. If ICH is confirmed, CT angiography is performed to assess the potential presence of cerebrovascular malformations or aneurysms. If ICH is absent, magnetic resonance imaging (MRI) is subsequently performed to explore alternative etiologies.

Although current understanding of ICH pathophysiology remains insufficient to manage the disease, advancements in neuroimaging techniques have improved diagnosis, evaluation, and management of the condition. HE is widely believed to be closely linked to poor outcomes in patients with ICH. Although current treatments targeting HE have shown limited benefits, early risk stratification using imaging can influence patient triage and monitoring intensity, potentially improving outcomes [95]. Consequently, early imaging is critical in managing patients with ICH. The diagnostic algorithm for ICH is shown in Figure 4.

One key imaging marker, the spot sign on CTA, often indicates active bleeding or rebleeding within the hemorrhage and is typically used as an eligibility criterion for hemostatic therapy trials in acute ICH [96]. This spot sign is associated with high mortality rates, poor neurological recovery, and great HE, as well as predicts ongoing bleeding during hematoma evacuation and postoperative rebleeding [96, 97, 98]. In patients receiving conservative treatment for spontaneous ICH, the spot sign is independently linked to high rates of HE and mortality and poor functional outcomes [98]. In those undergoing hematoma evacuation, the spot sign is associated with increased 90‐day mortality rates [99].

Meta‐analyses have shown that the spot sign can be detected in approximately one‐third of patients with ICH, with earlier imaging associated with higher detection rates and better predictive ability for HE [100]. While the spot sign is more common in intraventricular hemorrhage (IVH) than in parenchymal hemorrhage, it does not predict HE or clinical outcomes in IVH cases [101]. Additionally, including IVH expansion in the definition of HE does not affect the predictive value of CTA‐detected spot sign for significant HE. Although the spot sign seen on magnetic resonance imaging during the acute phase of ICH is associated with HE and poor outcomes, it has not yet been validated in large patient cohorts [102].

Retrospective analyses and subsequent independent validation have demonstrated that dual‐energy computed tomography (CT) has greater sensitivity and accuracy in predicting HE than traditional single‐energy CT, particularly when quantifying iodine content within the hematoma. Iodine leakage, detected using the gemstone spectral imaging technique of dual‐energy CT and known as the iodine sign, shows greater sensitivity and accuracy for early predicting HE and poor outcomes than the spot sign [103, 104, 105]. Delayed phase fusion imaging on dual‐energy CTA further increases sensitivity for predicting HE, from 32 to 76%, compared with traditional mixed arterial phase imaging. Moreover, the spot sign detected on delayed fusion images is independently associated with HE and poor outcomes [106].

## 6 Clinical Management

**FIGURE 5:**
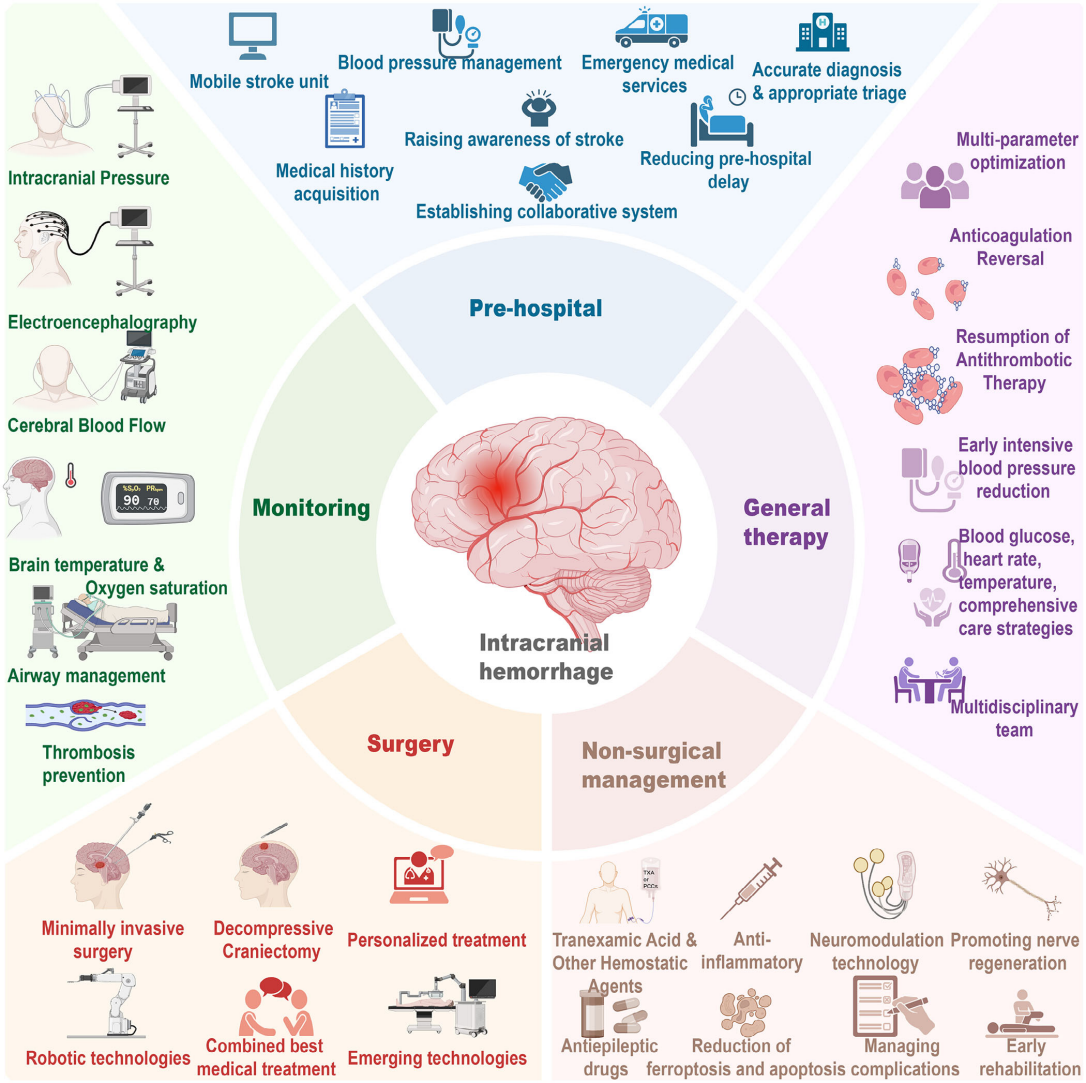
Key points in the management of intracerebral hemorrhage. For patients with ICH, comprehensive and continuous measures are required for clinical management. Before ICH onset, blood pressure should be well controlled. After ICH onset, symptoms should be quickly identified, and emergency transportation should be promptly arranged. After the patient is admitted to the hospital, relevant departments need to collaborate in a timely and efficient manner, and treatment approaches should be carefully selected. Each of these aspects has a significant impact on the patient's prognosis.

The section elaborates on a series of management measures covering the full course of care, from prehospital emergency care to in‐hospital treatment, specifically tailored to patients in the acute phase of ICH (Figure 5).

### 6.1 Prehospital and Emergency Care

In acute stroke care, time is a critical factor, and the feasibility of prehospital interventions is well established. Early intervention within the ultra‐acute phase, particularly within the first 2 h and even in the ambulance setting, is crucial for effective management of ICH [107]. This time window offers a key opportunity to administer time‐sensitive treatments. By applying the “time is brain” principle to ICH, optimizing and expanding prehospital management strategies can lead to significant improvements in patient outcomes [108, 109].

Accurate and timely diagnosis of ICH during the prehospital phase is crucial for effective triage and management [110]. Emerging noninvasive sensor technologies, along with mobile stroke units (MSUs) equipped with advanced imaging, hold significant potential to improve early detection and differentiation between ischemic stroke and ICH [111, 112, 113, 114]. Additionally, the use of prehospital biomarker sampling can expedite the identification of ICH, enabling the implementation of more precise and targeted interventions [115]. These technologies not only support early diagnosis but also help direct patients to the most appropriate treatment centers, thereby improving the overall efficiency of care. Although standardized assessment tool for accurate diagnosis is lacking, emerging portable stroke detection technologies—such as near‐infrared spectroscopy, ultrasound, electroencephalography, microwave technology, and impedance spectroscopy—show potential for improving prehospital triage and reducing delays [116]. Hospitals should prioritize optimizing triage procedures; shortening diagnostic turnaround times; and enhancing coordination between emergency, imaging, and surgical teams. Implementing standardized rapid assessment and intervention protocols can help minimize in‐hospital delays. By addressing existing inefficiencies and focusing on targeted improvements, healthcare systems can enhance timeliness and quality of care, ultimately leading to improved patient outcomes and reduced healthcare costs.

Hypertension is a major risk factor for poor outcomes in ICH, especially in patients on antithrombotic therapies, such as antiplatelets and anticoagulants [117]. Studies show that early, aggressive BP management during the prehospital phase can lower the risk of HE and improve survival rates [118, 119]. The link between prehospital systolic BP and ICH volume at admission further underscores the importance of early intervention [120]. While prehospital management offers significant benefits, it also presents challenges and potential risks. BP management during this phase should be tailored to the specific stroke subtype rather than applied uniformly. For patients with hemorrhagic stroke, lowering BP may reduce the risk of poor functional outcomes, but the approach may differ for patients with ischemic stroke [62]. Additionally, choosing the right antihypertensive agent is essential. Some studies suggest that using glyceryl trinitrate in the prehospital setting could lead to poor outcomes, particularly in patients with ICH [64, 65, 121]. Similarly, the effectiveness of magnesium sulfate and minocycline in treating acute ICH appears limited [73, 122, 123]. These findings highlight the potential for premature BP reduction or inappropriate pharmacological interventions to worsen brain injury, emphasizing the need for personalized treatment based on accurate diagnosis and patient‐specific conditions. While challenges remain, timely BP control, accurate diagnosis, and efficient triage are critical for improving care. Continued research and the integration of emerging technologies into prehospital care protocols are essential for improving ICH treatment and mitigating its devastating consequences.

### 6.2 General Supportive Therapy

#### 6.2.1 Anticoagulation Reversal and Resumption of Antithrombotic Therapy in ICH

ICH presents a substantial clinical challenge, particularly in anticoagulation reversal and the resumption of antithrombotic therapy [124]. Prompt reversal of anticoagulation in patients with ICH is crucial, as delays are associated with increased HE and mortality rates, especially among older individuals [125, 126]. Evidence shows that reducing the international normalized ratio (INR) to below 1.3 within 4 h of admission correlates with lower HE rates and improved outcomes [127]. The choice of reversal agent depends on the anticoagulant used. For patients on vitamin K antagonists, prothrombin complex concentrates and intravenous vitamin K are recommended to quickly restore INR levels. In contrast, specific reversal agents such as idarucizumab for dabigatran and andexanet alfa for factor Xa inhibitors are effective for patients on direct OACs [95]. However, limited availability and high costs of these agents remain barriers in some clinical settings. Current evidence supports the feasibility and effectiveness of resuming antithrombotic therapy (anticoagulation or antiplatelet therapy) after ICH, regardless of hemorrhage location or the presence of microbleeds [23, 128, 129, 130, 131, 132, 133, 134, 135]. For instance, the RESTART trial suggests that resuming antiplatelet therapy post‐ICH does not significantly increase the risk of recurrent hemorrhage and may reduce the occurrence of major vascular events [23]. Achieving a balance between preventing thromboembolic events and minimizing the risk of recurrent hemorrhage requires careful consideration of factors such as timing, the type of anticoagulant, and patient‐specific characteristics [136].

The decision to restart antithrombotic therapy following ICH is complex and demands a thorough assessment of the risks of thromboembolic events versus the potential for recurrent hemorrhage [137]. The timing of antithrombotic therapy resumption varies, with some studies suggesting that early reinitiation of antiplatelet therapy (within 1–2 weeks posthemorrhage) may benefit patients without HE and those with a history of cerebrovascular disease or chronic kidney disease [138, 139]. However, proponents of delayed therapy caution that early antiplatelet use may increase the risk of HE during the acute phase, particularly when the BBB remains compromised [140]. Whether to resume oral anticoagulation early (<2 weeks) or later (>4 weeks) depends on a careful evaluation aimed at reducing thrombosis risk while minimizing rebleeding [136, 140, 141, 142]. This decision should be made based on factors such as the cause of the initial hemorrhage, thromboembolic risk, and the presence of comorbidities. For instance, in patients with mechanical heart valves, restarting anticoagulation before day 13 post‐ICH may significantly increase bleeding risks, while day 6 could represent the earliest point at which thrombosis prevention benefits outweigh hemorrhage risks, particularly in those at a high thromboembolic risk [143]. In ICH patients with atrial fibrillation, long‐term OACs appear feasible, with left atrial appendage occlusion offering an alternative for those at a high risk of recurrent hemorrhage or with contraindications to anticoagulation therapy [22, 144, 145, 146]. The selection of post‐ICH antithrombotic therapy is critical, as different agents carry varying risks. Direct OACs seem to pose a lower ICH risk and offer better safety in recurrent bleeding scenarios than other antithrombotic agents, though variability exists across study outcomes, and debate in the field remains ongoing [147, 148, 149, 150, 151, 152].

Management of patients with ICH who require anticoagulation or antiplatelet therapy should involve a multidisciplinary team, including neurologists, hematologists, and cardiologists, to assess individual risks comprehensively [153]. Further research is needed to refine the timing and selection of antithrombotic therapy. Future studies should focus on stratifying patients based on genetic and biochemical markers to more accurately predict bleeding and thromboembolic risks. Additionally, developing new direct OAC reversal agents and optimizing existing therapies will be crucial in improving safety and outcomes for this vulnerable population.

#### 6.2.2 BP Management

Compared with patients with subarachnoid hemorrhage (SAH), those with spontaneous ICH often exhibit a more complex and multifactorial hypertensive response that is less reliant on catecholamines [154, 155]. Numerous studies emphasize the importance of early and aggressive BP control in reducing HE and improving clinical outcomes [11, 12, 156, 157, 158, 159, 160, 161]. The INTERACT2 trial is a landmark study demonstrating that intensive BP reduction is associated with improved functional outcomes at 90 days and tends to reduce mortality rates and severe disability [11]. However, other research highlights potential risks of overly aggressive BP reduction, including acute kidney injury and cerebral ischemia [63, 162]. In the hyperacute and acute phases, BP fluctuations, especially rapid swings in systolic BP, have been linked to poorer outcomes [163, 164, 165]. This emphasizes the need for maintaining stable BP levels, rather than simply targeting an absolute BP reduction.

In addition to differences between stroke subtypes [166, 167], factors such as age, imaging markers (e.g., “spot sign, ” microbleeds), perioperative status, prior BP management, time to achieving targeted systolic BP, and comorbidities all significantly impact the prognosis of acute‐phase BP control [168, 169, 170, 171, 172, 173, 174]. In patients with renal impairment or diabetes, overly aggressive BP reduction may increase the risk of ischemic complications, underscoring the need for individualized BP management strategies [175]. Recent advancements in BP management have focused on novel drugs and therapeutic approaches. Agents such as remifentanil, dexmedetomidine, nicardipine, and single‐pill combination therapies have proven effective in reducing BP in acute conditions with few side effects [69, 176, 177]. However, the choice of medication and administration route should be tailored to the patient's clinical condition and comorbidities, as different drugs may affect hematoma growth and functional recovery in varied ways [178, 179].

Long‐term BP control is essential for secondary prevention in ICH survivors. Research shows that inadequate BP management significantly increases the risk of lobar and nonlobar ICH recurrence, emphasizing the importance of strict control in preventing recurrent hemorrhage [180]. A comprehensive approach—including patient education, lifestyle modifications, and the use of combination antihypertensive therapy—is necessary to achieve rapid, effective, and sustained BP control [181, 182]. Current guidelines support a personalized strategy, focusing on immediate and long‐term BP management to improve outcomes. Future research should focus on refining these approaches, considering the diversity of ICH presentations and the expanding range of treatment options.

#### 6.2.3 Blood Glucose, Heart Rate, Temperature, and Comprehensive Care Strategies: Multiparameter Optimization

In ICH management, regulating blood glucose, heart rate (HR), and temperature has been widely studied and shown to significantly influence patient outcomes. A comprehensive approach that simultaneously optimizes these parameters could further improve clinical outcomes for patients with ICH.

Hyperglycemia is a common and critical metabolic disorder in ICH, often linked to poorer prognoses [183, 184, 185]. Stress‐induced hyperglycemia is a particularly strong predictor of adverse outcomes, with glucose level fluctuations associated with high mortality rates and unfavorable clinical outcomes [186, 187, 188]. Elevated glucose levels at admission has been closely associated with an increased risk of HE and poor prognosis, though outcomes vary between patients with and without diabetes [189, 190, 191]. In patients undergoing early thrombolysis, hyperglycemia raises the risk of symptomatic ICH, especially in older or severely affected individuals [192]. The pathological interactions between inflammation, sympathetic nervous system activation, and hyperglycemia may collectively worsen secondary brain injury [193, 194]. While some studies support the use of intensive insulin therapy to control glucose fluctuations and reduce neurological damage, its efficacy remains debated. Aggressive glucose management may lead to hypoglycemia, which itself increases the risk of ICH [195]. Further research is needed to balance the benefits of glucose control with its potential risks in ICH management.

HR and HR variability (HRV) are key physiological indicators of prognosis in patients with ICH [196]. An elevated HR is often linked to high mortality rates and poor functional outcomes, particularly during the acute phase [197]. Additionally, reduced HRV reflects autonomic nervous system dysfunction, indicating a poorer prognosis [198, 199]. Monitoring HRV offers valuable insights into disease progression and helps predict adverse outcomes [200, 201, 202]. Therefore, clinical management should focus not only on HR reduction but also on HRV changes to fully assess the patient's neurological status and prognosis.

Temperature management is also crucial in the care of patients with ICH in the acute phase. Fever is strongly associated with negative outcomes, including PHE expansion and increased mortality rates [203, 204]. Targeted temperature management, which involves maintaining normothermia or inducing mild hypothermia to reduce brain injury, has shown protective effects in various acute brain injury conditions [205, 206, 207, 208]. However, inconsistencies in defining fever, methods of temperature measurement, and treatment protocols make assessment of the full impact of post‐ICH fever or its treatment benefits challenging [209, 210]. Unlike its established role in cardiac arrest, targeted temperature management in acute stroke is less clearly defined and less frequently applied [211]. Additionally, the effects of targeted temperature management on long‐term functional outcomes should be further validate through RCTs.

Comprehensive care strategies, such as the intensive care bundle, focus on optimizing multiple physiological parameters simultaneously to improve patient outcomes through systematic nursing interventions. This approach prioritizes early intensive BP reduction while also managing hyperglycemia, fever, and anticoagulant disorders. Studies indicate that these care bundles significantly enhance functional outcomes at 6 months post‐ICH, with a lower incidence of adverse events compared with that in conventional care [14]. By integrating these management measures, comprehensive care strategies provide a multidimensional, patient‐centered model for ICH treatment, promoting the standardization and optimization of acute care, particularly in LMICs where resources may be limited.

### 6.3 Nonsurgical Management

#### 6.3.1 Tranexamic Acid and Other Hemostatic Agents

Tranexamic acid (TXA) is an antifibrinolytic agent commonly used in hemorrhagic conditions due to its ability to inhibit fibrinolysis and stabilize blood clots, making it a potential treatment option for ICH. However, its efficacy remains debated [212, 213]. The STOP‐AUST trial found TXA to be safe but not significantly effective in reducing HE [77], although secondary analysis suggested a modest, nonsignificant reduction in intraventricular HE [214]. Similarly, the TICH‐2 (TXA for hyperacute primary ICH) trial reported that while TXA reduced early mortality rates and serious adverse events, it did not significantly improve functional outcomes at 90 days [74]. One‐year follow‐up data from TICH‐2 showed no significant difference in functional recovery between TXA and placebo groups, though a slight improvement in survival was noted, suggesting that TXA may aid in prolonging survival without enhancing functional recovery [215]. Additionally, a TICH‐2 sub‐study demonstrated no significant increase in remote ischemic brain lesions with TXA use, indicating its favorable safety profile [216]. Nonetheless, TXA use in patients with conditions such as CAA may require caution, as these patients might be more vulnerable to adverse effects [216].

The “spot sign” has been proposed as a marker for identifying patients who could benefit from TXA. However, a subgroup analysis from the TICH‐2 trial revealed no significant difference in HE prevention between patients with and without the spot sign, suggesting it may not be sufficient for guiding treatment decisions [214]. Furthermore, timing appears to be a crucial factor, with earlier TXA administration potentially offering more benefits [217]. The STOP‐MSU trial investigated TXA use within 2 h of symptom onset but yielded unremarkable results [66]. Phase III trials are warranted to provide further clarity.

Recombinant activated factor VII (rFVIIa) has been extensively studied for its potential to limit HE in acute ICH. Early trials showed rFVIIa administered within 3 h of symptom onset could reduce hematoma growth and improve 90‐day outcomes [70], though its effectiveness depends on factors such as the presence of the spot sign and timing of administration [76, 218]. Data from the ANNEXA‐4 and TICH‐NOAC studies suggest that andexanet alfa significantly reduces the risk of HE compared with nonspecific treatments such as prothrombin complex concentrates and TXA. Importantly, andexanet alfa does not increase thrombotic complications, making it a safer option for high‐risk patients [83, 219].

Future research should focus on optimizing the timing and dosing of these agents, exploring their combination with other therapies, and identifying patient subgroups most likely to benefit. Additionally, the development of novel hemostatic agents with improved safety profiles and broader therapeutic windows could further enhance ICH management.

#### 6.3.2 Pharmacological Interventions

Antiepileptic drugs, such as levetiracetam, are frequently used to prevent post‐ICH seizures. However, research suggests that antiepileptic drugs do not significantly impact long‐term neurological outcomes, raising questions about their routine use for seizure prevention in patients with ICH [220, 221].

Inflammation may exacerbate the negative effects of anemia on patient outcomes, and targeted neuroinflammatory therapies, including antibody, cell, and gene therapies, may offer new treatment possibilities for patients with ICH [222, 223]. Anti‐inflammatory strategies have shown promise, with drugs including the interleukin (IL)‐1 receptor antagonist Anakinra, fingolimod, statin, and celecoxib showing potential in reducing PHE and modulating inflammatory responses [224, 225, 226, 227, 228, 229, 230, 231, 232, 233]. However, their effectiveness in improving functional outcomes remains under investigation. Minocycline, a MMP‐9 inhibitor, has also demonstrated neuroprotective potential in reducing secondary brain injury in ICH [73, 234, 235, 236]. Further validation is needed, particularly in prehospital settings or when used adjunctively with hematoma evacuation surgery. Iron overload‐induced neurotoxicity, which disrupts the BBB and leads to cell death through ferroptosis and apoptosis, has gained increasing attention. Deferoxamine mesylate has emerged as a promising neuroprotective agent due to its ability to reduce iron‐mediated oxidative damage. Early trials have shown that deferoxamine mesylate is safe, but its impact on significantly improving functional outcomes remains inconclusive [75]. However, exploratory analyses suggest that deferoxamine may hold potential, particularly in enhancing long‐term outcomes for patients with moderate‐volume ICH (10–30 mL), warranting further research into its role in ICH management [237, 238].

Stem cell therapy, especially using mesenchymal stem cells (MSCs), has also garnered interest for its regenerative potential in ICH [239, 240, 241]. Preliminary studies indicate that MSCs may promote neurogenesis and reduce inflammation, potentially aiding in functional recovery, though large‐scale trials are needed to confirm their efficacy and safety [242, 243, 244]. In patients with antiplatelet‐associated ICH, desmopressin has been shown to improve platelet function and may reduce the risk of HE [82, 245, 246]. However, its routine use should be approached with caution, as it may lead to adverse effects such as hyponatremia. Current evidence suggests that platelet and red blood cell transfusions should not be routinely used but rather considered on a case‐by‐case basis [72, 247].

Statins, due to their pleiotropic effects, have been considered for use in patients with ICH. While concerns about an increased risk of recurrent ICH persist, current data indicate that statins do not significantly increase the risk of major bleeding events [231, 232, 248]. Other agents, including vitamin D, certain antidiabetic drugs such as glyburide, and reactive oxygen species scavengers, have shown promise in ICH treatment, though translation into clinical practice remains a challenge [80, 249, 250, 251, 252].

### 6.4 Surgical Interventions

While traditional craniotomy has long been the standard approach, its effectiveness has been a topic of debate [253]. The STICH and STICH II trials showed that early surgery did not significantly improve functional outcomes [84, 85]. In recent years, significant advancements have been made in the surgical treatment strategies for ICH, particularly with the introduction of minimally invasive surgery (MIS) and other emerging technologies.

The MISTIE trial evaluated the safety and efficacy of MIS combined with recombinant tissue plasminogen activator in patients with ICH. The findings suggested that MIS with recombinant tissue plasminogen activator may offer advantages in improving functional outcomes [86]. Subsequent analyses indicated that reducing ICH volume to ≤15 mL or by ≥70% improves long‐term outcomes [254]. The MISTIE III trial established a residual hematoma volume of ≤15 mL as a surgical target. While MIS effectively reduced hematoma volume and showed potential in lowering early mortality rates, it did not significantly improve functional outcomes at 1 year, possibly due to delays in reaching the surgical target or slow hematoma drainage [87].

Recent studies comparing traditional craniotomy, MIS, and conservative treatment have shown that MIS, particularly endoscopic surgery and stereotactic aspiration, holds promise for improving patient outcomes [88, 255, 256, 257, 258, 259, 260, 261]. Patients who underwent surgery within 24 h of ICH onset, especially for lobar hemorrhage, had better functional outcomes at 180 days and lower 30‐day mortality rates compared with those receiving medical therapy alone [90]. Despite these findings, challenges remain with MIS, including technique refinement, optimal timing, patient selection, adjunctive therapies, and equitable access. In cerebellar ICH, surgical decisions may depend on hematoma size, and surgery may be suitable for patients at a high risk of seizures [262, 263]. The combination of deferoxamine mesylate with MIS shows potential but currently lacks sufficient supporting evidence [264].

Decompressive craniectomy (DC), aimed at relieving ICP, has shown mixed results across different types of ICH. The SWITCH trial compared DC with optimal medical treatment, reporting that DC may offer benefits for certain patients, though it did not significantly reduce severe disability or death overall [89]. DC may not be suitable for all cases, and conservative treatment could be preferable in some instances [91]. Early findings suggest that robot‐assisted surgery could enhance safety and outcomes, and as technology advances, its role in ICH treatment is expected to grow [265, 266].

Despite the challenges in ICH surgical treatment, recent studies support the use of MIS and new technologies. These studies have expanded our understanding of surgical approaches and offered insights into optimizing patient selection and timing. Future research should explore integrate MIS, pharmacological therapies, and robotic technologies to achieve optimal outcomes for patients with ICH.

### 6.5 Monitoring and Intensive Care Unit Management

Effective management of ICH relies heavily on precise monitoring of ICP, cerebral perfusion pressure, and other neurophysiological parameters. Clinical research indicates that cerebral perfusion pressure levels in early hemorrhagic stroke do not significantly correlate with mortality rates, whereas elevated ICP is strongly associated with higher 28‐day intensive care unit (ICU) mortality rates, and maintaining ICP below 16.5 mmHg may be critical for survival [267]. Thus, ICP may require more attention in early treatment decisions. The SYNAPSE‐ICU study found that ICP monitoring significantly reduced mortality rates at 6 months, particularly in severe cases, such as in those with nonreactive pupils [268]. However, while ICP monitoring may improve short‐term survival, it does not seem to impact long‐term functional outcomes [269]. Maintaining stable ICP during MIS for ICH has also been shown to aid recovery by providing real‐time feedback, allowing immediate intervention if ICP rises and potentially reducing complications such as rebleeding [270].

Despite the safety of ICP monitoring in clinical practice, its effect on long‐term functional outcomes remains unclear. Patients undergoing ICP monitoring often receive aggressive treatment, but those with elevated ICP still face poor long‐term outcomes despite intervention. A recent meta‐analysis indicated that while continuous ICP monitoring may reduce short‐term mortality rates in some ICH subgroups, it does not improve neurological outcomes at 6 months [271]. Additionally, variability of ICP is emerging as an area of interest, with rapid ICP fluctuations linked to an increased risk of HE, warranting further investigation through RCTs [272]. Noninvasive ICP monitoring methods, such as measuring optic nerve sheath diameter via ultrasound, are gaining attention for their safety and ease of use. While optic nerve sheath diameter offers a promising alternative to invasive methods, the lack of standardized thresholds for elevated ICP limits its broader application [273].

Monitoring brain temperature is also critical in ICH treatment, especially in the presence of cortical spreading depolarizations [274]. Early detection and management of elevated brain temperature may reduce secondary brain injury and improve outcomes [275]. Cautious use of supplemental oxygen in patients with ICH is recommended, especially in the acute phase. While oxygen therapy prevents hypoxemia, excessive oxygen can cause adverse effects [276]. For instance, the SETPOINT2 study found that early tracheostomy in patients with stroke requiring mechanical ventilation did not significantly improve 6‐month outcomes compared with standard tracheostomy timing, possibly due to excessive oxygen administration [276, 277].

Cerebral blood flow (CBF) and metabolic activity monitoring are equally important in ICH care, and electroencephalogram (EEG) monitoring aids in detecting seizures and tracking disease progression [278]. Transcranial doppler with quantitative EEG (QEEG) has been proposed as an effective method to predict prognosis in patients with severe acute ICH. Transcranial doppler helps indirectly measure ICP, whereas QEEG reflects the relationship between CBF and cerebral metabolism. Studies have shown that combining these techniques significantly improves the accuracy of predicting 90‐day mortality rates compared with using either method alone [279].

These findings highlight the potential of multimodal monitoring for comprehensive brain function assessment and guiding treatment. However, further research is needed to refine these technologies, establish standardized thresholds, and determine their impact on long‐term recovery. The future of ICH management lies in developing multimodal monitoring strategies tailored to individual patient needs, ultimately improving survival and quality of life.

### 6.6 Delays in Care and Systems Challenges

Although imaging technologies facilitate early identification of ICH, diagnostic imaging investigations are often performed too late due to various unexpected delays. Therefore, implementation of early intervention is strongly recommended to prevent stroke. Identifying and addressing the delays in advance is crucial for reducing complications, slowing down disease progression, and improving outcomes. Delays are generally categorized as prehospital delay (the time from symptom onset to initial medical consultation) and in‐hospital delay (the time from emergency department arrival to final treatment) [280]. (The “final treatment” refers to evidence‐based interventions for ICH implemented by skilled medical professionals in the hospital setting).

Factors contributing to prehospital delays include patients' or bystanders' lack of awareness and barriers to accessing emergency services; long travel distance to medical facilities; patient age; wake‐up strokes; disease severity; and family structure [281, 282, 283, 284, 285, 286]. Prehospital transfer protocols can significantly influence functional outcomes and mortality in patients with ICH. Evidence suggests that direct transfers to higher‐level stroke centers, while intended to provide superior care, are associated with increased rates of complications and mortality compared with transfers to the nearest stroke center [287]. These findings underscore the importance of minimizing prehospital delays and initiating emergency treatment as early as possible. They also challenge the traditional preference for delayed but higher‐quality care, suggesting that earlier appropriate treatment may yield better outcomes. In‐hospital delays are often attributed to inefficiencies in the triage process, delays in examinations, and poor coordination among medical teams. Misdiagnosis is another key factor, making magnetic resonance imaging a priority for patients with mild strokes or symptoms persisting for a week or more [288, 289, 290].

Both prehospital and in‐hospital delays can lead to complications such as HE, increased ICP, hospital‐acquired infections, and secondary hemorrhagic transformation, all of which contribute to poor overall outcomes [287, 291]. The relationship between the time from symptom onset to treatment and discharge outcomes is thought to be nonlinear, with a critical “threshold” value. For instance, in patients with SAH, this threshold is 12.25 h; beyond this, the benefits of treatment decrease, and the risk of death rises sharply after 24 h [292]. Each additional day of treatment delay further extends hospital stays by an average of 2 days, increasing stroke‐related healthcare costs, particularly among older adult patients [288]. Moreover, prolonged waiting times and uncertainty can heighten emotional distress, negatively affecting the overall patient experience [293].

Despite the critical role of delays, studies investigating the critical impact of delays remain insufficient, with some studies suggesting that early arrival does not significantly affect outcomes [280, 294, 295]. However, focusing solely on short‐term outcomes provides a limited perspective and hinders the development of effective strategies to reduce delays.

## 7 Neural Repair and Recovery

**FIGURE 6:**
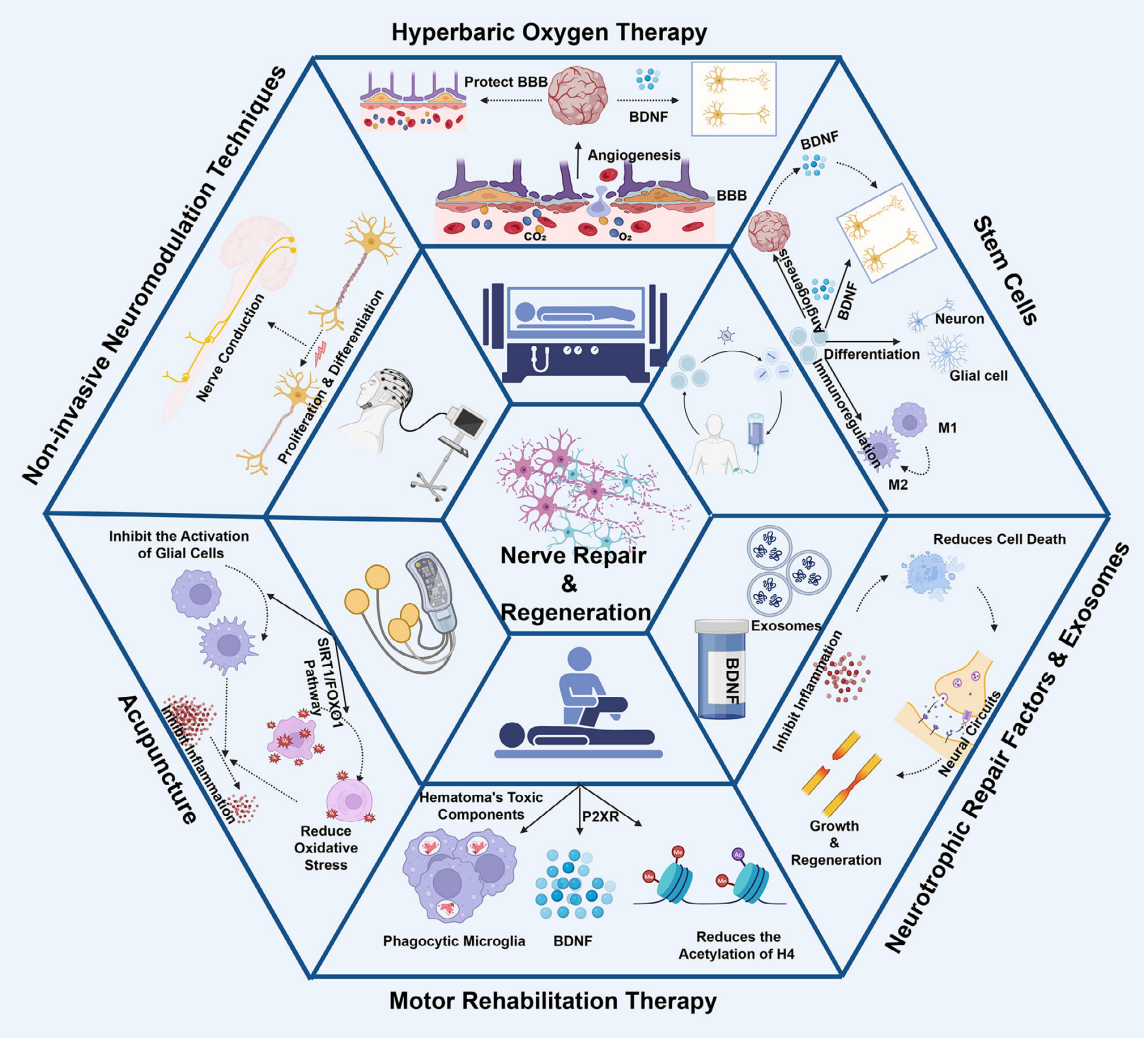
Neural regeneration and functional recovery strategies for intracerebral hemorrhage. ICH triggers a series of events including neuronal apoptosis, synaptic dysfunction, and the impairment of neuronal self‐repair, leading to a decline in motor and cognitive abilities. Currently, stem cell therapy, neurotrophic factors, and rehabilitation therapy have been proven to repair damaged neurons, promote reconstruction of neural networks, and assist in functional recovery by regulating angiogenesis, microglial polarization, and neuroinflammation.

Despite timely clinical intervention, most patients still experience varying degrees of sensory dysfunction. This is because when ICH occurs, the neurons responsible for regulating various motor and cognitive functions are damaged, thereby affecting the structure and function of the brain. Specifically, a series of problems, such as neuronal apoptosis, synaptic dysfunction, and disruption of the neurons’ self‐repair process, may arise. Once neurons are damaged, the brain's normally well‐orderly functional operations are disrupted, leading to declines in the motor and cognitive abilities. However, with effective neuroprotective and repair measures, damaged neurons may be restored, promoting reconstruction of the neuronal network and gradual recovery of functions. To improve prognosis and promote effective recovery of neurological function in patients with cerebral hemorrhage, the following section comprehensively discusses the neural repair strategies following ICH including drug therapy, cell therapy, rehabilitation training, and physical therapy (Figure 6). Additionally, we have summarized outstanding research findings from the past decade in Table 2, organized according to their mechanisms of action.

**TABLE 2:** Preclinical research.

Animal model	Year	Intervention	Result (treatment/control)	References
Autologous blood injection ICH rat	2015	Autologous bone marrow‐derived mononuclear cells	A reduction in neutrophil infiltration, a decrease in the number of inducible nitric oxide synthase‐positive cells, a decline in the expression of inflammation‐related signals, a mitigation of brain edema in the peri‐hematoma area, an increase in vascular density and migration ability were noted. Moreover, improvements were noted in motor function recovery, alleviation of spatial learning and memory impairment, and a reduction in brain atrophy.	[296]
Collagenase‐induced ICH rat	2016	UC‐MSCs	Intracerebral and intravenous administration of UC‐MSCs are mainly distributed around the hematoma, and both can significantly improve the neurological function of rats with ICH and reduce the volume of brain injury.	[297]
Autologous blood injection ICH rat	2017	BMSCs	BMSC transplantation may alleviate neurological deficits and activate axonal regeneration by increasing the expression of GAP‐43 through the activation of ERK1/2 and PI3K/Akt signaling pathways.	[298]
Collagenase‐induced ICH rat	2018	MSCs	The mortality rate, hematoma size, and the count of degenerative neurons were significantly reduced, while the expression of endothelial junction proteins was increased.	[299]
Collagenase‐induced ICH mice	2019	ASCs	It alleviates brain edema and inflammation, reduces cell apoptosis, and improves the functional outcomes after ICH.	[300]
Collagenase‐induced ICH rat	2020	Human umbilical cord Wharton's jelly‐derived MSCs	The treatment reduced the residual hematoma volume in rats with moderate ICH, but not severe ICH.	[301]
Collagenase‐induced ICH mice	2021	Hypoxia‐preconditioned olfactory mucosa MSCs	It reduced the activity of microglia and the expression of IL‐1β and TNF‐α. Furthermore, it reduced the pyroptosis‐related proteins around the hematoma, decreased the expression of microglial NLRP3 and caspase‐1, and reduced the membrane pores of microglia.	[302]
Chronic ICH rat	2022	BMSCs	Higher potential for secreting nutritional factors, better cell proliferation ability, better neural recovery, higher expression of BDNF, less brain atrophy.	[303]
Collagenase‐induced ICH mice	2023	MSCs	Improve inflammation‐induced neurological deficits and brain water content by inhibiting the Mincle/Syk signaling pathway	[304]
ICH‐induced injury in vitro	2024	OM‐MSCs	LncRNA CAPN15, lncRNA ALDH1L2, mir‐3473b, and mir‐19643p are upregulated in thrombin‐stimulated OM‐MSCs, while GM20431, lncRNA GAPDH, and mir‐122b‐3p are downregulated.	[305]
Autologous blood injection ICH mice	2025	OM‐MSC	IGF2BP2 enhances the protective effect of OM‐MSCs against ICH‐induced brain injury. IGF2BP2 stimulates the neuronal differentiation of OM‐MSCs through the SENP1/IGF2BP2/SOX11 axis.	[306]
Collagenase‐induced ICH rat	2019	GDNF transduction to MSCs	The transplanted MSCs‐GDNF survive 2 weeks after transplantation; express NSE, MAP2, and GFAP; and secrete bioactive compounds (BDNF, NGF‐β), which significantly improve neurological function.	[307]
Autologous blood injection ICH rat	2018	MANF	Significantly increasing the ratios of p‐Akt, p‐MDM2, and Bcl/Bax; reducing the expression of p53 and caspase‐3, as well as neuronal death; and improving neurological function.	[308]
Autologous blood injection ICH rat	2019	Multipotent MSC‐derived exosomes	A significant increase in newly formed endothelial cells in the hematoma border zone, neuroblasts and mature neurons in the subventricular zone, and myelin in the striatum, but the lesion volume remained unchanged.	[309]
Collagenase‐induced ICH mice	2021	Exosomes from miR‐19b‐3p‐modified ASCs	Effectively attenuates heme‐induced cell damage and ferroptosis, and significantly improves the neurological function.	[310]
Collagenase‐induced ICH rat	2023	BDNF	The concentration of BDNF in the CSF was higher than that of the control. Blocking BDNF reduced the promoting effect on the proliferation and differentiation of neural stem cells.	[311]
Collagenase‐induced ICH rat/mice	2023	rhCDNF	rhCDNF inhibits proinflammatory cytokine production, enhancing erythrophagocytosis, and increasing anti‐inflammatory mediators. It then upregulates the Nrf2–HO‐1 pathway in microglia/macrophages, alleviating peri‐hematoma oxidative stress.	[312]
Collagenase‐ induced ICH mice	2023	Exogenous administration of miRNA‐124‐overexpressed microglial exosomes	Genetically edited exosomes can alleviate neurological deficits and brain edema, improve the integrity of the BBB, and reduce nerve cell death. Moreover, the protective effect is abolished in mice lacking Gr‐1⁺ bone marrow cells.	[313]
Autologous blood injection ICH mice	2023	Exosomes derived from young healthy human plasma	Exosomes are mainly distributed around the hematoma and can be internalized by neuronal cells. They significantly enhance the behavioral recovery by reducing brain damage and cellular ferroptosis.	[314]
Collagenase‐induced ICH mice Hemoglobin‐treated astrocytes	2024	ADEVs	Astrocytes activated by OGD reduce heme‐induced hyperpermeability of endothelial cells by secreting EVs and alleviate the severity of ICH in both in vivo and in vitro experiments.	[315]
Collagenase‐induced ICH mice	2025	Loaded with pEGFP encoding human BDNF	Improved the neurological function, and alleviate acute cerebral edema and neuroinflammation	[316]
Collagenase‐induced ICH rat Hemoglobin‐treated astrocytes	2025	hUCMSC‐ex	Downregulates TLR4, NF‐κB/P65, and p‐P65; reduces the proinflammatory cytokines TNF‐α and IL‐1β; and increases the expression of anti‐inflammatory cytokine IL‐10, thereby improving the behavioral functions of rats.	[317]
Collagenase‐induced ICH rat	2016	Treadmill exercise	DNA fragmentation and the expressions of caspase‐3 and Bax decreased, whereas the expressions of 5‐HT and tryptophan hydroxylase increased. Apoptosis was reduced, and motor function was improved.	[318]
Autologous blood injection ICH Rat	2019	HBO	HBO treatment downregulates M1 marker proteins, while the levels of M2 marker proteins remain unchanged. The expression of proinflammatory cytokine decreases, and neuronal death and neurological deficits are alleviated.	[319]
Collagenase‐induced ICH mice	2021	Treadmill Exercise	The hematoma volume decreased, and the number of phagocytic microglia increased. Soluble factors increased in the plasma, including endostatin, IGFBP‐2 and 3, and MMP‐9.	[320]
Collagenase‐induced ICH mice	2023	HBO	HBO alleviated neuronal injury and neurological function recovery in ICH rats and reduced serum S100β and NSE content.	[321]
Autologous blood injection ICH rat	2021	Acupuncture	Acupuncture alleviates neuronal cell death, inflammation, and ferroptosis after ICH by downregulating miR‐23a‐3p.	[322]
Autologous blood injection ICH rat	2024	Acupuncture	Acupuncture reduced oxidative stress injury and neuronal apoptosis via activating SIRT1/FOXO1 pathway. The neuroprotective effects of acupuncture were abolished by injection of the SIRT1 inhibitor EX527.	[323]

Abbreviations: 5‐HT, 5‐hydroxy‐tryptamine; ADEVs, astrocyte‐derived extracellular vesicles; ASCs, adipose‐derived stem cells; BDNF, brain‐derived neurotrophic factor; CNTF, ciliary neurotrophic factor; CSF, cerebrospinal fluid; EVs, extracellular vesicles; FBD, fibrin‐binding domain; GDNF, glial cell line‐derived neurotrophic factor; HBO, hyperbaric oxygen; hUCMSC‐ex, human umbilical cord mesenchymal stem cells exosomes; ICH, intracerebral hemorrhage; BMSCs, bone marrow stromal cells; IGFBP, insulin‐like growth factor‐binding protein‐2 and ‐3; MMP, matrix metallopeptidase‐9; IL‐1β, interleukin‐1 beta; TNF‐α, tumor necrosis factor‐α; MANF, astrocyte‐derived neurotrophic factor; MMPs, matrix metalloproteinases; NLPR3, NLR family, pyrin domain containing protein 3; NSC, neural stem cells; OGD, oxygen‐glucose deprivation; OM‐MSCs, olfactory mucosal mesenchymal stem cells; rhCDNF, recombinant human cerebral dopamine neurotrophic factor; ROS, reactive oxygen species; UC‐MSC, umbilical cord tissue‐derived mesenchymal stromal cells.

### 7.1 Stem Cell Therapy and Neurogenesis

MSCs have been garnering increasing attention within the realm of nerve repair and regeneration due to their unique advantages. One of the remarkable features of MSCs is their low immunogenicity, which enables them to integrate seamlessly into both autologous and allogeneic systems [324]. This characteristic significantly reduces the likelihood of immune‐related complications, making them an ideal candidate for therapeutic applications. MSCs have a diverse spectrum of functions. They exhibit potent immunomodulatory properties that can fine‐tune the immune response to create a favorable microenvironment for tissue repair. Through the secretion of extracellular vesicles, MSCs initiate self‐recovery processes. These vesicles are laden with a rich cargo of bioactive molecules that can stimulate cell survival, proliferation, and restoration of damaged tissues [325, 326]. Moreover, MSCs are capable of performing regenerative repair, effectively restoring injured neural tissues [327]. When transplanted into damaged brain tissue, stem cells can differentiate into specific neural cells under the induction of the local microenvironment, replacing damaged or dead neurons and glial cells, which helps maintain the integrity of neural conduction pathways, reconstruct neural circuits, and improve neural function [328]. Additionally, stem cells can secrete a various neurotrophic factors, such as brain‐derived neurotrophic factor (BDNF), nerve growth factor (NGF), and glial cell‐derived neurotrophic factor (GDNF). These factors can promote the survival, growth, and differentiation of neural cells; inhibit neural cell apoptosis; promote the growth of nerve axons and the formation of synapses; and create a favorable microenvironment for nerve repair [329, 330, 331]. In addition, MSCs can effectively inhibit or regulate the immune responses triggered by the complex interactions among immune cells such as T lymphocytes, B lymphocytes, dendritic cells, and NK cells [325]. Some studies have confirmed that MSCs can polarize proinflammatory M1 phenotypes into anti‐inflammatory M2 phenotypes through CX3C chemokine receptor 1 (CX3CR1). Downregulating CX3CR1 can significantly alleviate microglial activation and peripheral inflammatory responses; reduce serum IL‐6, IL‐1β, and TNF‐α levels; promote the differentiation and maturation of mature oligodendrocytes; and alleviate myelin dysfunction caused by brain injury [332, 333]. In ICH mouse models, after the transplantation of MSCs, astrocytes undergo astrocyte–mesenchymal phenotype transformation, proliferate by downregulating phosphorylated macrophage stimulatory protein and phosphorylated‐yes‐associated protein, and protect cells from apoptosis [334]. The transplantation of bone‐derived MSCs (BMSCs) can increase the level of GFAP, a biomarker of astrocytes, and improve the astrocyte–mesenchymal phenotype transformation and antiapoptotic ability by upregulating the expression level through the Cx43/Nrf2/HO‐1 axis [335]. Based on these characteristics, current research shows that stem cells can reshape neurons through neurotrophic and inflammatory regulation, repair tissue damage, and positively affect the treatment of nerve damage after ICH [336]. BMSCs are commonly used in the treatment of brain injuries, as they are easy to obtain from the host and can infiltrate the damaged brain area through the BBB without destroying its structure [328]. They are the most studied stem cells and are considered promising candidates for regenerative medicine. For instance, BMSCs have been shown to limit neurological deficits and BBB dysfunction in rats with ICH [337]. In addition to bone marrow‐derived sources, MSCs from human umbilical cord, umbilical cord blood, placenta, amniotic fluid, amnion, and adipose tissue have also been proven to promote the differentiation of neuron‐like and astrocyte‐like cells around the damaged area, increase the expression level of vascular endothelial growth factor, and restore neural function [297, 338, 339]. However, although animal studies of MSC treatment for ICH have provided strong evidence, showing that MSC transplantation in ICH mice confers anti‐inflammatory and angiogenic effects, which can significantly reduce cognitive, motor, and hematoma‐related functional impairments, the risk of tumor formation remains an important safety concern [340]. Moreover, although preclinical studies have shown positive outcomes, such as reduced degree of tissue damage, inflammation, oxidative stress, and reversal of neurodegeneration markers, the therapeutic effects of MSC‐based treatments in clinical studies are still under investigation [341]. By 2017, stem cell therapy for ICH had advanced to RCTs. A double‐blind RCT for ICH sequelae showed improvements in the ability of daily living (modified Barthel index) and functional independence scores of patients in the MSC group, with no serious adverse reactions reported [17]. Although the sample size was not large, this was the first positive signal from an RCT for stem cell treatment in ICH. Due to the low immunogenicity and abundant availability of induced‐pluripotent stem cells (iPSCs), as well as the ability to overcome the ethical limitations associated with embryonic stem cells, research in the following years focused on reprogramming patients’ somatic cells to obtain iPSCs and then differentiating them into neural progenitor cells [342]. However, iPSC therapy in the field of cerebral hemorrhage is still in the early stage of exploration. Nevertheless, future research should “custom‐make” autologous neural cells for each patient to repair the brain tissue defects caused by hemorrhage. In 2024, a preliminary phase I, dose‐escalating, safety, and tolerability trial using human BMSCs in patients with ICH confirmed that the therapy was well‐tolerated within the given dose range, and no serious adverse reactions occurred [244]. These findings lay the foundation for subsequent large‐scale controlled studies.

In conclusion, although positive signals indicate the potential of stem cell treatment for ICH, this therapy is still in the early stage of clinical research. Currently, most trials are phase I safety trials or small‐sample phase II exploratory trials, and no large‐scale randomized controlled phase III trials have been published to date. Therefore, there is still a long way to go before the relevant therapy can be applied in routine clinical practice. Compared with ischemic stroke, the number and scale of stem cell clinical trials for ICH are limited, which further hinders the clinical translation of stem cell therapy.

### 7.2 Neurotrophic Factors and Exosomes

Neurotrophic repair factors play a crucial role in the neural repair following ICH. BDNF promotes neuronal survival, growth, and differentiation and enhances synaptic plasticity [329]. It can activate downstream signaling pathways such as the tropomyosin‐related kinase B receptor‐related signaling pathway, regulate neuronal gene expression and intracellular signal transduction, and strengthens neuronal resistance to injury [343]. After ICH occurs, an increase in BDNF levels in the local microenvironment can provide nutritional support for damaged neurons, guide the growth and extension of nerve axons, and promote the reconstruction of neural circuits [343]. Relevant studies have shown that BDNF supplementation improved functional recovery in ICH rat models [344]. A positive correlation exists between the expression level of BDNF mRNA in the ipsilateral cortex of the affected limb after ICH and motor function [345]. NGF mainly acts on sympathetic and sensory neurons. It can promote the growth and regeneration of nerve fibers, maintain the survival and function of neurons, and help reconnect damaged nerve fibers and restore nerve conduction function after ICH [346, 347, 348]. GDNF has specific protective and nutritional effects on dopaminergic neurons and motor neurons. It can promote metabolic and functional recovery of neurons, reduce the apoptosis of neurons, and has potential therapeutic value for improving motor dysfunction after ICH [307, 349].

Exosomes are nanoscale vesicles secreted by cells. They carry a large number of specific substances such as proteins (e.g., integrin proteins, immunomodulatory proteins, transmembrane proteins, exosome‐producing proteins), mRNA, noncoding RNA, DNA, and lipids [350]. As an important medium for intercellular communication, they have great potential in the field of neural repair. Compared with stem cells, exosomes have advantages such as the ability to freely cross the BBB, convenient storage and transportation, high biological safety, low immunogenicity, and small immune rejection reactions [351]. In the treatment of ICH, exosomes contribute to neural repair through various pathways. Moreover, they can inhibit inflammatory responses, reduce programmed cell death, protect the BBB, and alleviate oxidative stress damage. After treating ICH mice with the exosome inhibitor GW4869, the number of inflammatory cells infiltrating the periphery increased significantly, and the expression levels of IL‐1β and IL‐6 increased, indirectly proving that exosomes can inhibit the neural inflammatory response after ICH [352]. Exosomes can also transport miRNA‐126, miRNA‐125a, and miRNA‐124 to promote angiogenesis, neural plasticity, myelin formation, and neurite remodeling, thereby promoting neural functional recovery [353, 354, 355].

### 7.3 Rehabilitation Strategies

Patients with ICH usually experience varying degrees of deficits in movement, sensation, recognition, language, and perception. In addition to poor balance and limited mobility, they often experience weakness, stiffness, and changes in movement patterns. Many physical therapy strategies focus on helping patients recover quickly. Guiding patients to actively engage in rehabilitation training during the recovery period is of positive significance for accelerating their rehabilitation process. As a noninvasive clinical treatment approach, motor rehabilitation therapy focuses on improving sensorimotor function and promoting recovery of neurological function. Both clinical trial data and animal models have shown that motor rehabilitation can significantly enhance motor recovery after ICH, reduce the disability rate, and improve the quality of life [356, 357, 358]. A 6‐week pretreatment of treadmill exercise effectively promoted recovery of neurological dysfunction triggered by ICH in mice [320]. This pretreatment not only reduced lesion volume but also boosted the quantity of phagocytic microglia [320]. Conversely, aerobic exercise, such as the 6‐week treadmill exercise, can stimulate the production of BDNF, thereby enhancing learning and memory abilities in mice. [359]

Hyperbaric oxygen therapy (HBO) can increase the partial pressure of oxygen in blood and damaged tissues by increasing the dissolved oxygen content, improving cerebral oxygenation, and increasing oxygen metabolism. It can deliver oxygen to the damaged area of the brain tissue, improve cerebral microcirculation, and promote vascular endothelial cell regeneration in the damaged brain tissue [360]. Early initiation of HBO can prevent the activation of MMP‐9 and the destruction of endothelial junction proteins, reduce cerebral edema and BBB damage, as well as reduce neuroinflammation by regulating microglial activation after ICH, thus improving nerve cell function and behavioral deficits [361, 362].

Noninvasive neuromodulation techniques, such as transcutaneous vagus nerve stimulation (tVNS), repetitive transcranial magnetic stimulation (rTMS), and transcranial direct‐current stimulation, have been proven to improve the motor function in patients with stroke and are becoming increasingly recognized in stroke rehabilitation [363]. These techniques are worthy of in‐depth exploration as early intervention strategies for the treatment of ICH. For instance, after rTMS treatment in ICH mice, the proliferation of neural stem cells and neuronal differentiation were obvious, whereas glial cell differentiation was weakened [364]. Experiments utilizing the ICH rat model has been demonstrated that acute phase tVNS treatment can provide neuroprotection, limit HE, and improve neurological function after ICH [365]. However, current research on noninvasive neuromodulation techniques mainly focuses on ischemic stroke, and relevant research on neural recovery in hemorrhagic stroke needs to be strengthened.

### 7.4 Acupuncture

Electroacupuncture is applied in neurological rehabilitation owing to its unique mechanism of inhibiting the activation of glial cells and reducing the release of inflammatory substances. Specifically, it can downregulate CX3CL1 and increase IL‐10 (an anti‐inflammatory cytokine), thereby decreasing the release of inflammatory substances such as proinflammatory cytokines [366]. Relevant studies have revealed the efficacy and mechanism of electroacupuncture and acupuncture in the treatment of ICH from experimental and clinical aspects. Experiments conducted on rat ICH models have confirmed that acupuncture can improve neurological deficits and cerebral edema after ICH and alleviate pathological damage and neuronal degeneration in the peri‐hematoma area [366]. Acupuncture has also been shown to activate the silent mating type information regulation 2 homolog‐1 (SIRT1)/forkhead box protein O1 pathway, thereby reducing oxidative stress injury and neuronal apoptosis. When the SIRT1 inhibitor EX527 is administered, the neuroprotective effect of acupuncture is reduced. In addition, electroacupuncture may play a neuroprotective role in acute ICH through the caveolin‐1/MMP/BBB permeability pathway [323]. Clinical research on CT outcomes in patients with ICH have suggested that acupuncture can improve hematoma clearance rate, promote contraction and absorption of hematomas, and reduce physical damage to brain tissue [367]. In particular, scalp acupuncture can reduce the levels of TNF‐α, C‐reactive protein, IL‐1, and IL‐6 after cerebral infarction, thereby interrupting the inflammatory response storm, protecting neurological function, and promoting recovery following ICH [368].

Although previous studies have demonstrated the potential of electroacupuncture and acupuncture in treating ICH, current research remains limited and insufficiently explored, with a lack of comprehensive global research coverage.

## 8 Prognostication and Predictive Models

ICH is a highly heterogeneous cerebrovascular disease, with prognosis closely associated with various factors [369, 370]. Early identification and intervention to prevent HE are essential for improving patient outcomes. Current evidence has identified several key predictors of HE [371, 372, 373, 374, 375].

### 8.1 Imaging Predictors

Noncontrast CT markers, such as hypodensity, the “black hole sign, ” and mixed density, along with derived imaging indicators including the frequency of imaging markers, have been extensively studied for their predictive value in HE and clinical outcomes [376, 377, 378, 379, 380, 381, 382]. Additionally, the presence of diffusion‐weighted imaging positive lesions may indicate an increased risk of recurrent ICH and predict poor functional outcomes [383, 384, 385]. While these imaging biomarkers are effective in identifying high‐risk patients, further research is needed to confirm their utility in guiding specific therapeutic strategies.

PHE is another common imaging finding following ICH, and its growth is strongly associated with poor outcomes [386]. Studies indicate that the volume and growth rate of PHE are often predictive of high mortality rate and poor functional outcomes, particularly in the acute phase [387, 388]. Additionally, the imaging characteristics of PHE, such as shape and volume changes, are critical indicators of prognosis, as irregular shapes may reflect more complex or severe pathological changes beyond BBB disruption and cytotoxic edema [389, 390, 391, 392]. In cases with highly heterogeneous edema morphology, the peak edema extension distance may provide a more accurate prognosis than PHE volume alone [393].

Some studies suggest that changes in ventricular size may more accurately predict clinical outcomes in patients with ICH than with PHE [394]. The presence and rapid expansion of IVH are linked to high acute mortality rates and significantly poor long‐term functional outcomes [395, 396]. IVH is also associated with nonlobar hematomas and an increased risk of early neurological deterioration, indicating that incorporating IVH assessment could enhance our understanding of ICH pathophysiology and improve risk stratification strategies [397, 398]. While no method currently exists to quantify the rate of ventricular blood clearance, diffusion tensor imaging may be used for its evaluation [399]. Hematoma location is another crucial factor influencing ICH prognosis, with clinical outcomes depending on hematoma volume in specific locations [400, 401]. Patients with deeper brain hemorrhages generally have poorer functional outcomes, even after adjustments for confounding factors [400]. In contrast, while patients with lobar hemorrhages may have large hematomas, their prognosis can improve if HE is effectively controlled [402]. However, survivors of lobar ICH are likely to experience uncontrolled hypertension at 6 months after the event, which may explain the high recurrence risk in patients with frontal lobe hemorrhages observed in other studies [403].

### 8.2 Biological Factors

Physiological factors such as BP, blood glucose, and HR play a key role in predicting outcomes for patients with ICH [186, 189, 197, 404, 405, 406, 407]. Additionally, other factors including age, laboratory tests (including lipid levels, blood counts, and inflammatory markers), nutritional status, skin sympathetic nerve activity, and dynamic functional connectivity can help identify high‐risk patients and guide timely interventions, warranting further research [408, 409, 410, 411, 412, 413, 414, 415, 416, 417, 418].

Sex‐based differences in clinical presentation, treatment response, and overall prognosis are notable among patients with ICH [419, 420]. A meta‐analysis revealed that male patients tend to present with larger hematomas and have higher mortality rates within 3 months compared with female patients [421]. While women generally have a lower incidence of ICH, advanced age and more severe symptoms contribute to higher long‐term mortality risk [422]. Race and ethnicity also affect prognosis, with African American and female patients showing less‐efficient functional recovery [423]. In contrast, Asian patients, particularly those receiving BP‐lowering treatments, exhibit better outcomes, including reduced HE risks and mortality rates [424]. Patients in high‐altitude regions, however, tend to experience more severe symptoms and worse short‐term outcomes than those living in low‐altitude regions, highlighting the importance of controlling BP, oxygen levels, and inflammation in such settings [425].

Multimorbidity is closely linked to prior disability and significantly impacts short‐term mortality after stroke [426]. For instance, untreated hypertension is associated with high in‐hospital mortality rates, with racial differences playing a vital role [427, 428]. Comorbidities such as sepsis, early seizures, systemic inflammatory response syndrome, and stroke‐associated pneumonia are associated with increased short‐term mortality and poor long‐term outcomes in patients with ICH [429, 430, 431, 432].

The growing recognition of brain–heart interactions, particularly the occurrence of stroke–heart syndrome, underscores the need for further exploration of cardiovascular complications after ICH. Research shows that new cardiovascular complications, known as stroke–heart syndrome, frequently develop after ICH and are strongly linked to poor 5‐year outcomes [433]. Similar attention is needed for neuro‐nephrology, which explores the interactions between the brain and kidneys, but these areas require further investigation [434].

### 8.3 Others

The potential clinical utility of thromboelastography markers in ICH needs further investigation, particularly regarding their universality and underlying mechanisms, to determine whether viscoelastic hemostatic assay‐guided treatment should be integrated into ICH care; similarly, the use of optic nerve ultrasound also warrants further investigation [435, 436, 437].

Incorporating the aforementioned factors is essential for developing personalized treatment strategies, improving survival rates, and enhancing the quality of life for patients with ICH. Future research should continue to explore these mechanisms and optimize care for diverse patient populations.

## 9 Knowledge Gaps and Future Directions

Despite advances in medical science, ICH remains associated with high mortality and morbidity rates, and significant knowledge gaps persist in its research.

### 9.1 Pathophysiological Mechanisms

Following ICH, a complex immune response process is initiated within the brain. Specifically, peripheral immune cells infiltrate intracranial tissues, while resident cells undergo shifts in their polarization states. These intricate immune interactions form a precise and complex regulatory network that determine neuronal fates, leading to apoptosis or initiation of repair processes. Notably, the changes in the intracranial immune state induced by ICH are remarkably persistent and are likely to last for decades. However, the intrinsic relationship between the immune activation state and neural injury and repair remains incompletely understood. In recent years, multiple studies have achieved remarkable results in addressing the oxidative stress response caused by toxic components in hematomas. The newly discovered drainage pathway of the brain‐to‐cervical lymphatic nodes (CLNs), as an intracranial substance clearance channel, has been proven to accelerate the clearance of intracranial hematomas in ICH mice models and effectively preserve neural function [438, 439]. This discovery provides new potential targets and treatment strategies for ICH. Nevertheless, several challenges remain in translating the results of animal experiments into clinical practice, and the road to clinical translation is still long. In the future, researchers need to make more efforts to conduct more in‐depth and systematic studies to elucidate the mechanism of action of the brain‐to‐CLN drainage pathway, bringing new breakthroughs and hopes for the treatment of patients with ICH.

### 9.2 Diagnostic and Imaging Technologies

Current imaging technologies often fail to capture the dynamic changes occurring in the brain post‐ICH, such as hematoma growth, BBB disruption, and ischemic progression. Numerous imaging biomarkers with potential prognostic value have been identified, but their diagnostic and predictive capabilities remain insufficiently studied. The development, validation, and integration of these biomarkers could enhance clinical decision‐making. Personalized management of BP and glucose levels, anticoagulation reversal, and antithrombotic therapy following ICH are essential. Optimizing multiple parameters simultaneously may offer new avenues for improving outcomes and should be a focus of future research, though considerable challenges persist. Moreover, the application of artificial intelligence (AI) in the diagnosis of ICH remains in its early stages. Further research is urgently needed to verify AI's ability to accurately diagnose ICH and precisely predict disease progression. However, with improvements in algorithmic performance, AI‐assisted diagnosis holds great promise in clinical practice and can significantly reduce the burden on healthcare providers.

### 9.3 Therapeutic and Translational Challenges

Despite existing challenges, the effective translation of biomarker‐ and multiomics‐based approaches from the laboratory to clinical practice is crucial for disease diagnosis, etiological classification, and severity stratification. Early prehospital diagnosis, severity assessment, and ultra‐early intervention are of paramount importance. Identifying and preemptively addressing prehospital and in‐hospital delays, as well as integrating new technologies into prehospital care protocols, should be the ongoing research priorities.

### 9.4 Clinical Trial Design and Implementation

#### 9.4.1 Clinical Management

Surgical management of ICH requires careful consideration of technique, timing, patient selection, adjunctive therapies, and equitable access. Future research should continue to explore the integration of MIS, pharmacological therapies, and robotic technologies to achieve optimal treatment outcomes for patients with ICH. In addition to precisely determining the timing of surgical interventions and reasonably selecting the surgical approach, two methods hold promise for promoting local hemostasis and thereby reducing acute injuries. One is the use of drug synergy to enhance hemostatic effect and the other is the administration of drugs locally through the surgical access. However, rigorous clinical studies are still required to verify their effectiveness and safety.

Currently, consensus on best practices for continuous monitoring of patients with ICH is lacking. The interpretation of brain monitoring device data and its impact on treatment decisions are not yet fully established. Developing comprehensive monitoring systems that integrate multiple modalities, such as ICP, CBF, and EEG, could provide a more holistic view of brain health and better guide therapeutic interventions.

#### 9.4.2 Secondary Prevention

Approximately 5% of patients with ICH experience recurrence annually [440]. Although appropriate BP control is widely recognized to confer greater benefits for the secondary prevention of ICH than of ischemic stroke, several crucial questions remain unanswered. Whether intensive BP control poses potential risks to vital organs such as the heart and kidneys, and whether the BP control targets are consistent across different subtypes of patients with ICH is yet to be determined. Moreover, for patients with atrial fibrillation or those who have been on long‐term antiplatelet therapy, further RCTs are required to establish the optimal timing for resuming anticoagulation therapy and to evaluate the safety of anticoagulation regimens. Similarly, additional evidence is needed to confirm that healthy lifestyle habits, including smoking cessation, diabetes management, regular exercise, and a low‐salt diet, are beneficial for the secondary prevention of ICH.

### 9.5 Global Health and Disparities

The highly heterogeneous and multifactorial nature of ICH, including its incidence and treatment outcomes, have not been thoroughly studied. Establishing global surveillance systems is essential to identify regional risk factors and guide targeted prevention strategies, particularly in LMICs.

## 10 Limitations of Current Evidence

Although we have spared no effort to comprehensively summarize the pathological mechanisms and clinical management strategies of ICH, the limitation of the article's length makes it difficult to provide a meticulous and in‐depth interpretation of every aspect. In particular, the mechanism of action of peripheral immune cells after ICH, considering the large number of intricate and intertwined cell signaling transduction pathways involved, could not be explored in‐depth. Moreover, this review mainly focuses on the pathological mechanisms and management methods of the acute phase of ICH, with limited coverage of the chronic recovery phase. In addition, the scope of this study is restricted to ICH, with little attention given to SAH and IVH. Although SAH and IVH are intrinsically related to ICH, they substantially differ in pathological mechanisms, clinical manifestations, and treatment approaches. The lack of exploration of these types of hemorrhages may limit readers’ broader understanding of ICH and reduce the applicability of the research findings to clinical practice. In view of this, future research should adopt a broader perspective and incorporate other hemorrhagic subtypes to provide a more comprehensive and in‐depth reference for clinical practice and future studies.

## 11 Conclusion

ICH remains a formidable challenge in clinical practice due to its high mortality and morbidity rates. Addressing these challenges through continued research and the development of tailored therapeutic approaches is crucial to reduce the global burden of ICH and improve patient's quality of life.

The concept of “time is brain, ” originally established in the treatment of acute ischemic stroke, is not only applicable but also relevant to the treatment of acute ICH. For patients with acute ICH, hemostatic treatment should be initiated within the first few hours of disease onset. The earlier implementation of prehospital and MSU technologies to initiate hemostatic treatment can considerably increase the likelihood of functional recovery and societal reintegration.

Individualized and precise adjustment of clinical treatment plans facilitates the diagnosis and treatment of the disease. However, accurately and promptly determining the optimal intervention timing for patients with ICH has always been a challenging task. This calls for the collaborative efforts of multiple disciplines, including neuroimaging, molecular biology, and symptomatology, to precisely predict the progression of the disease after ICH.

Encouragingly, the increasing number of preclinical studies and clinical trials on ICH provides continued hope for reducing the burden of this devastating disease.

## Author Contributions

TL and RJ conceptualized the study. TL, MZ, and WJ prepared and drafted the manuscript. TL, MZ, WJ, XC, and SX performed the literature search. and TL, MZ, WJ, and LC created figures. All authors have read and approved the final version of the manuscript.

## Conflicts of Interest

The authors declare no conflicts of interest.

## Data Availability

The authors have nothing to report.
